# High aspect ratio nanomaterial-induced macrophage polarization is mediated by changes in miRNA levels

**DOI:** 10.3389/fimmu.2023.1111123

**Published:** 2023-01-27

**Authors:** Johanna Samulin Erdem, Táňa Závodná, Torunn K. Ervik, Øivind Skare, Tomáš Hron, Kristine H. Anmarkrud, Anna Kuśnierczyk, Julia Catalán, Dag G. Ellingsen, Jan Topinka, Shan Zienolddiny-Narui

**Affiliations:** ^1^ National Institute of Occupational Health, Oslo, Norway; ^2^ Department of Genetic Toxicology and Epigenetics, Institute of Experimental Medicine, the Czech Academy of Sciences, Prague, Czechia; ^3^ Institute of Molecular Genetics, Academy of Sciences of the Czech Republic, Prague, Czechia; ^4^ Department of Clinical and Molecular Medicine, Norwegian University of Science and Technology, Trondheim, Norway; ^5^ Proteomics and Modomics Experimental Core Facility and St. Olavs Hospital Central Staff, Trondheim, Norway; ^6^ Department of Work Safety, Finnish Institute of Occupational Health, Helsinki, Finland; ^7^ Department of Anatomy, Embryology and Genetics, University of Zaragoza, Zaragoza, Spain

**Keywords:** macrophage, polarization, nanomaterials, inflammation, fibrosis, epigenetic, miRNA

## Abstract

**Introduction:**

Inhalation of nanomaterials may induce inflammation in the lung which if left unresolved can manifest in pulmonary fibrosis. In these processes, alveolar macrophages have an essential role and timely modulation of the macrophage phenotype is imperative in the onset and resolution of inflammatory responses. This study aimed to investigate, the immunomodulating properties of two industrially relevant high aspect ratio nanomaterials, namely nanocellulose and multiwalled carbon nanotubes (MWCNT), in an alveolar macrophage model.

**Methods:**

MH-S alveolar macrophages were exposed at air-liquid interface to cellulose nanocrystals (CNC), cellulose nanofibers (CNF) and two MWCNT (NM-400 and NM-401). Following exposure, changes in macrophage polarization markers and secretion of inflammatory cytokines were analyzed. Furthermore, the potential contribution of epigenetic regulation in nanomaterial-induced macrophage polarization was investigated by assessing changes in epigenetic regulatory enzymes, miRNAs, and rRNA modifications.

**Results:**

Our data illustrate that the investigated nanomaterials trigger phenotypic changes in alveolar macrophages, where CNF exposure leads to enhanced M1 phenotype and MWCNT promotes M2 phenotype. Furthermore, MWCNT exposure induced more prominent epigenetic regulatory events with changes in the expression of histone modification and DNA methylation enzymes as well as in miRNA transcript levels. MWCNT-enhanced changes in the macrophage phenotype were correlated with prominent downregulation of the histone methyltransferases *Kmt2a* and *Smyd5* and histone deacetylases *Hdac4*, *Hdac9* and *Sirt1* indicating that both histone methylation and acetylation events may be critical in the Th2 responses to MWCNT. Furthermore, MWCNT as well as CNF exposure led to altered miRNA levels, where miR-155-5p, miR-16-1-3p, miR-25-3p, and miR-27a-5p were significantly regulated by both materials. PANTHER pathway analysis of the identified miRNA targets showed that both materials affected growth factor (PDGF, EGF and FGF), Ras/MAPKs, CCKR, GnRH-R, integrin, and endothelin signaling pathways. These pathways are important in inflammation or in the activation, polarization, migration, and regulation of phagocytic capacity of macrophages. In addition, pathways involved in interleukin, WNT and TGFB signaling were highly enriched following MWCNT exposure.

**Conclusion:**

Together, these data support the importance of macrophage phenotypic changes in the onset and resolution of inflammation and identify epigenetic patterns in macrophages which may be critical in nanomaterial-induced inflammation and fibrosis.

## 1 Introduction

Environmental and occupational pulmonary exposures may lead to inflammation and fibrosis in the lung. Understanding the cellular and molecular mechanisms regulating the onset of acute inflammatory responses and development of chronic inflammation and fibrosis is important. In these processes macrophages play pivotal roles as they are involved not only in the onset but also the resolution of inflammatory responses. The macrophage population is highly heterogeneous and has a high phenotypic plasticity in response to environmental cues. The intricate population of macrophages cannot be easily characterized, and while it is generally accepted that the phenotype is dynamic, the traditional M1/M2 phenotype classification is still commonly used as an outline to assess inflammatory responses (1). Similar to environmental pollutants, inhaled nanomaterials may lead to inflammatory responses in the lung and if left unresolved, poorly soluble and biopersistent materials, e.g., high aspect ratio nanofibers, may bioaccumulate and induce chronic inflammation and fibrosis in the lung and pleura (2, 3). In the resolution and transition of immune responses, a timely alteration of macrophage phenotypes is imperative. Albeit various nanomaterials have been shown to possess immunomodulating properties, the involvement of macrophage polarization in nanomaterial-induced pulmonary inflammation and fibrosis is not well understood. The transition of macrophage phenotypes is tightly controlled by transcriptional and metabolic changes and is fine-tuned by epigenetic regulation (1, 4, 5). The epigenetic regulation occurs through histone modifications (e.g., methylation and acetylation mostly at lysine residues), DNA (5mC) and RNA modifications (e.g., m6A, m5C, m1A, 2’-O-Me, and Y), and non-coding RNAs. Together these epigenetic events allow alterations in gene transcription and translation by changing the promoter accessibility and destabilizing target transcripts important for macrophage functions. In the reprogramming of macrophages in response to external stimuli, histone modifications have been most extensively studied, and histone modifications at enhancers or promoters of inflammation-related genes are heavily altered by epigenetic enzymes (6). These enzymes, i.e., histone methyltransferases (HMTs) and demethylases (HDMs), acetyltransferases (HATs) and deacetylases (HDACs), are responsible for adding and removing histone modifications and therefore dictate the magnitude and type of immune response. Methylation of histones can result in either gene activation or repression depending on the site. Methylation of histones H3K4, H3K36, and H3K79 are commonly associated with gene activation while H3K9, H3K27, and H4K20 marks indicate gene silencing. HMTs are commonly associated with M2 macrophage activation by repressing M1 genes and promoting M2 gene transcription, while HDMs are generally linked to induction of M1 phenotype (5). Furthermore, certain histone acetylation marks have been shown to contribute to macrophage phenotypes. H3 acetylation, specifically H3K9 and H3K14, is important for M1 phenotypes. These sites are modified by HAT and HDAC activity, and while the role of HATs has not been thoroughly investigated, extensive evidence suggest a role of HDACs in macrophage polarization (7, 8).

There is also compelling evidence that non-coding RNAs play pivotal roles in the fine-tuning of macrophage phenotypes and resolution of inflammation. Non-coding RNAs e.g., miRNAs exert transcriptional and post-transcriptional regulation of inflammatory pathways. Several studies have confirmed that many different miRNAs affect macrophage polarization (reviewed in 9, 10). Furthermore, recent RNome work by Ma et al. identified several miRNAs differentially expressed during M1 and M2 polarization (9). Although, the regulation is complex and affected by many factors such as species differences, macrophage population and surrounding microenvironment, miR-21, miR-26a, miR-27a and b, miR-155 as well as miR-125a, miR-146a and b, and let-7c have been identified as critical regulators of M1 and M2 polarization (9–11). The involvement of DNA and RNA modifications in the regulation of macrophage polarization is less well known. While DNA methylation may influence macrophage activity and the DNA methyltransferases DNMT1 and DNMT3b, are important in the regulation of macrophage polarization (12–14), the role of RNA modifications is unstudied. As the biologic roles of RNA modifications are starting to emerge, it is however becoming increasingly evident that these dynamic modifications represent a new layer of control of genetic information.

Although changes in macrophage polarization have been implicated in the inflammatory and fibrotic responses to nanomaterials, the regulation of their immunomodulating properties needs further investigation. As such nanocellulose (NC) materials are both inflammogenic and immunomodulating, and NC exposure induces inflammation in the lung (15–21), increases the secretion of proinflammatory cytokines and chemokines, e.g., IL1B, IL1RA, IL6, IL8, TNFA, MCP1, CCL3, CCL4, CSF2, and GCF3 (22–26), and enhances M1 macrophage phenotype (27). However, the inflammatory response is timely resolved, and NC exposure does not result in pulmonary fibrosis (17, 28). In contrast, multi-walled carbon nanotubes (MWCNT) may, like asbestos fibers, induce pulmonary fibrosis and mesothelioma in exposed animals (2, 3). The onset of MWCNT-induced fibrosis is characterized by Th2-type responses following the initial acute inflammation, which is manifested as an induction in e.g., IL4, IL5 and IL13 in the BAL and the lung of exposed animals (29–33) as well as elevated levels of IL4 and IL5 in sputum of MWCNT-exposed workers (34), and is suggested to involve macrophage phenotypic changes (35–38). To evaluate the immunomodulatory effects of these two classes of industrially relevant nanomaterials, we here utilized a murine alveolar macrophage model which was exposed to cellulose nanocrystals (CNC), cellulose nanofibers (CNF) and two MWCNT under air-liquid interphase conditions. Furthermore, the potential contribution of epigenetic regulation in nanofiber-induced macrophage polarization was investigated by assessing the regulation of epigenetic regulatory enzymes, miRNAs, and rRNA modifications following exposure.

## 2 Materials and methods

### 2.1 Particle preparation and characterization

CNC (12.1%) was purchased from University of Maine Process Development Centre, ME, USA. CNF (1.0%) produced at Aalto University, Finland, was a kind gift from Prof. H. Norppa, Finnish Institute of Occupational Health, Finland. In addition, the two JRC MWCNT materials NM-400 (JRCNM04000a) and NM-401 (JRCNM04001a) were used. NC dispersions were prepared in sterile ultrapure water and the MWCNT were dispersed in 0.05% Bovine Serum Albumin (BSA; m/v in H_2_O). CNC dispersions were vortexed for 30 s and CNF and MWCNT dispersions were sonicated using a probe sonicator at 10% amplitude (Sonifier 450S, Branson Ultrasonics, Danbury, CT, USA) for 16 min. Prior to nebulization, MWCNT dispersions were passed through a 40 µm filter to remove large agglomerates. Endotoxin levels were assessed by kinetic chromogenic limulus amebocyte lysate (LAL) assay according to the manufacturer’s instructions (Lonza, Basel, Switzerland). Endotoxin levels of CNC and MWCNT particles were below the detection limit of 0.005 EU/ml. Endotoxin content in CNF was measured to 0.14 EU/ml. Hydrodynamic diameter was measure for the crystalline particle (CNC) by dynamic light scattering (DLS) (ZetaSizer Nano ZS, Malvern Instruments Ltd, Malvern, UK). Scanning electron microscope (SEM) specimens of nebulized samples were prepared on newly cleaved poly-L-lysine-coated mica, essentially as previously described (27). In brief, the specimens were sputter-coated with 2.4 nm platinum in a Cressington 208HR (Cressington Scientific Instruments, Watford, UK) sputter coater and analyzed with a Hitachi SU 6600 (Hitachi High-Technologies Corporation, Tokyo, Japan) field emission scanning electron microscope (FE-SEM). The instrument was operated under the following conditions: accelerating voltage of 15-20 kV and a working distance of 10 mm. High resolution images of the particles were obtained by acquiring at slow scanning speed. Length and diameter of the nanomaterials were measured using ImageJ software (39). At least 150 fibers/particles were measured for each material. Curved nanofibers were measured using the Simple Neurite Tracer plugin in Image J (40).

### 2.2 Air-liquid interface cell exposure

Murine alveolar macrophages, MH-S (CRL-2019, ATCC, Rockville, MD, USA) were maintained in ATCC-formulated RPMI-1640 medium (Gibco, ThermoFisher Scientific, MA, USA) supplemented with 10% ultra-low endotoxin FBS (Biowest, Nuaillé - France), and 50 μM β-mercaptoethanol (Gibco, ThermoFisher Scientific) in 5% CO_2_ at 37°C. MH-S cells were seeded in Falcon cell culture inserts (PET membranes, 4.2 cm^2^ growth area, 0.4 μm pore size, 1.6 × 10^6^ pores/cm²; Corning, NY, USA) at a concentration of 1.0E^6^ cells/well. The cells were allowed to attach overnight and were air-lifted immediately prior to exposure. Air-lifted cell cultures were exposed at air-liquid interface (ALI) using Cloud 6 (Vitrocell, Waldkirch, Germany) to nanoparticles at concentrations C1: 0.15 μg/cm^2^ and C2: 2.7 μg/cm^2^. Cells exposed to the dispersant were used as controls. Exposure experiments were performed in duplicates and repeated three times. Aerosol generation was performed by Aeroneb 4.0 - 6.0 μm nebulizers for CNC and Aeroneb 10 μm nebulizers for CNF and MWCNT dispersions. Shortly, the dispersions were adjusted with 0.01% NaCl to optimize nebulization efficiency, and 200-1000 μl dispersion was nebulized to obtain the desired deposited doses measured by the Quartz Crystal Microbalance (QCM). The measured deposited doses were 0.20 ± 0.04 μg/cm^2^ and 2.4 ± 0.3 μg/cm^2^ for CNC, 0.19 ± 0.04 μg/cm^2^ and 2.5 ± 0.1 μg/cm^2^ for CNF, 0.14 ± 0.03 μg/cm^2^ and 2.8 ± 0.3 μg/cm^2^ for NM-400, and 0.14 ± 0.03 μg/cm^2^ and 2.8 ± 0.4 μg/cm^2^ for NM-401. After exposure, cells were transferred to i) culture media (M0), ii) media with IFNG (20 ng/ml; PeproTech, NJ, USA), or iii) media with IL4 and IL13 (20 ng/ml each; PeproTech). IFNG-stimulated and IL4/IL13 stimulated cells are hereafter denoted as M1 and M2 cells, respectively. Polarized MH-S macrophages have been previously thoroughly characterized (27). M1 (IFNG) and M2 (IL4/L13) polarization was confirmed on air-lifted MH-S cells, **Supplementary Figure 1**. A simplified classification of macrophage M1 and M2 phenotype was performed by analyzing the expression of classical M1 and M2 makers by qPCR. The classical M1 markers assessed included the proinflammatory cytokine (*Il6*), nitric oxide synthase (*Nos2*), and Th1-cell attracting chemokines (*Cxcl9* and *Cxcl10*). The M2 phenotype was characterized by assessing the expression of receptors required for phagocytosis and scavenging of mannose (*Mrc1*, encoding CD206), markers involved in the arginase pathway (*Arg1*), as well as Th-2 cell response chemokines (*Ear11*). Four and 24h post-exposure, aliquots of cell media were collected for analysis of cytokine/chemokine and LDH release, and 24h post-exposure cells were collected for nanoparticle uptake, viability analysis and RNA extraction.

### 2.3 Uptake and cell viability

Uptake of nanomaterials was analyzed by transmission electron microscopy (TEM) at the Electron Microscopy Lab, Oslo University, Norway. In short, 24h after exposure cells were fixed with double strength PHEM fix (41), postfixed in 1% OsO_4_ (Electron Microscopy Sciences, PA, USA) and stained with 1% UA (Electron Microscopy Sciences). The specimens were dehydrated in an ethanol series, embedded in EPON (Sigma Aldrich, MO, USA) which was polymerized at 60°C and ultrathin sections (80 nm) were made with a Leica ultramicrotome (Leica Microsystems, Wetzlar, Germany). For visualization of NC materials, the sections were labeled with a biotinylated carbohydrate binding module (CBM) of β-1,4-glycanase (EXG : CBM) which was a kind gift from Dr H. Wolff (Finnish Institute of Occupational Health, Finland) and Prof. U. Vogel (National Research Centre for Work Environment, Denmark) (42). In brief, the sections were incubated with 1% fish skin gelatin for 30 min and washed twice with 0.1% BSA-PBS. The NC materials were stained using the biotinylated EXG : CBM protein at 1:500 dilution in 1% BSA-PBS for 30 min. Samples incubated with 1% BSA-PBS for 30 min instead of the EXG : CBM protein were used as negative controls. The EXG : CBM protein was visualized by immunogold labeling. Accordingly, the washed sections were incubated with a rabbit anti-biotin antibody (ab53494, Abcam, Cambridge, UK) at 1:300 dilution in 1% BSA-PBS for 30 min, followed by repeated washing in 0.1% BSA-PBS and incubation with 10 nm protein A gold (Cell Microscopy Core, UMC Utrecht, the Netherlands) at 1:50 dilution for 30 min. The stained sections were washed and allowed to air dry. All incubations were performed at room temperature. Images were taken in a JEOL 1400plus TEM equipped with a Ruby camera at 120 kV (JEOL Ltd., Tokyo, Japan). Cell viability and proliferation were assessed by acridine orange DAPI live dead discrimination using Via1-Cassette on a NucleoCounter NC-200 instrument (ChemoMetec, Allerod, Denmark), and cell membrane leakage was analyzed by CyQUANT™ LDH Cytotoxicity Assay (ThermoFisher Scientific), according to the manufacturer’s instructions. In the LDH analysis, lysed cells were included as a positive control indicating the maximum LDH release (100% LDH release), furthermore, a negative control for the spontaneous release of LDH was included corresponding to 0% LDH release. These controls were utilized in the calculation of LDH release according to the manufacturer’s recommendations.

### 2.4 RNA extraction and RT-qPCR analysis

Total RNA was extracted using RNA/DNA Purification Kit and RNase-Free DNase I Kit (Norgen Biotek Corp., Ontario, Canada). Purity and concentration were assessed by Nanodrop 2000 spectrophotometer and Qubit fluorometric measurement (ThermoFisher Scientific). Gene expression was analyzed by RT-qPCR using SYBR Green I technology on a QuantStudio 5 Real-Time PCR System (Applied Biosystems, ThermoFisher Scientific). For assessment of macrophage polarization, RNA was reverse transcribed using qScript cDNA synthesis kit, according to the manufacturer’s instructions (Quanta BioSciences, MA, USA). Primer sequences (KiCqStart™ Primers, Sigma Aldrich) are available in **Supplementary Table 1**. Expression of genes encoding epigenetic modification enzymes was assessed by a custom RT2 array (Qiagen, Hilden, Germany), order information is available upon request. Expression was normalized to the geometric mean of *Ubc* and *Hprt* (for individual assays) and *Hprt*, *Tbp* and *Ubc* (for the RT2 array). Expression was assessed using the ddCt method.

### 2.5 Analysis of cytokine and chemokine secretion

Concentrations of mouse cytokines/chemokines were measured in culture media by Bio-Plex Pro™ Mouse Cytokine 23-plex, according to manufacturer’s instructions (Bio-Rad Laboratories Ltd, CA, USA). CCL3 was excluded from the analysis as the samples fell outside of the standard curve.

### 2.6 miRNA sequencing and differential expression analysis

miRNA libraries were prepared using QIAseq miRNA library kit (Qiagen) and QIAseq miRNA NGS 96 Index IL according to the manufacturer’s instructions. Library concentrations were measured by Qubit 4.0 fluorometer using dsDNA HS assay kit (Invitrogen, ThermoFisher Scientific). The size and purity of the libraries were evaluated on Agilent 5200 Fragment Analyzer System using HS NGS Fragment Kit (1–6000 bp) (Agilent Technologies, CA, USA). For sequencing, the libraries were pooled at an equimolar concentration and denatured according to the standard Illumina NextSeq Library pooling guide. Sequencing was performed on Illumina NextSeq 550 system using NextSeq™ 500/550 High Output Kit v2.5 (75 cycles) (Illumina, CA, USA) following the manufacturer’s instructions. For detection and quantification of miRNAs in sequencing data the miARma-Seq v1.7.2 toolset was used (43). Specifically, adapters were trimmed from raw reads using CutAdapt and sequences between 18-26 bp and average quality >25 Phread score were included in further analysis. The sequences were mapped to the mouse reference genome (GRCm38) using Bowtie1 with the following parameters: –seedlen 19 –seedmms 0 –best –nomaqround. Reads mapped to miRNA regions annotated in miRBase Release 22.1 were counted using the featureCounts tool (44). Differential expression analysis of detected miRNAs was performed with the DEseq2 v1.36.0 tool using default parameters (45). miRNAs with less than 10 reads in total across all samples were excluded. CNF and NM-401-treated samples were compared to control groups for M1 and M2 cells respectively. Differentially expressed miRNAs with False Discovery Rate, FDR < 0.1 were considered as statistically significant. Variance stabilizing transformation was performed on raw count data prior Principal Component Analysis. Heatmaps were generated using a heatmap tool included in NMF v0.17.6 R package. Before plotting, raw count data were RPKM-normalized and log-transformed. Color scale in heatmaps represents row-normalized Z-scores. Volcano plots were generated using the EnhancedVolcano v1.14.0 R package (46). In the volcano plots a cutoff of FDR ==0.1 were utilized, this corresponds to the plotted uncorrected p-values of -log10(p-value)==2.5 and to -log10(p-value)==2.0 in M1 and M2 cells, respectively. mRNA targets of differentially expressed known miRNAs were estimated using miRDB v6 web service (47, 48). Only validated sets of functional miRNAs (the FuncMir Collection in miRDB) were considered. Genes with target prediction score <60 or more than 2000 predictions were excluded. Predicted target genes of significantly differentially expressed miRNAs were then used for statistical overrepresentation test in PANTHER Pathways v17.0 (49). It should be noted that the results obtained from the pathway analysis relies both on the prediction of differentially expressed miRNAs, and on the consequent miRNA target prediction. To minimize potential accumulative error effects, previously outlined filtering thresholds were applied in each step. Whole set of mouse genes was used as a reference set for Fisher’s Exact test. Results with False discovery rate < 0.05 were plotted using the ggplot2 R package.

### 2.7 Quantification of RNA modifications by LC-MS/MS

rRNA was extracted from total RNA using an Agilent 1260 Infinity II Analytical-Scale LC-UV Purification System with a Bio SEC-3 300 Å, 2.1 x 300 mm column (Agilent Technologies) chromatographed isocratically with 100 mM ammonium acetate pH 7 at 0.280 ml/min and 40°C for 20 min. Chromatograms were recorded at 260 nm and peaks corresponding to 18S and 28S rRNA were collected, lyophilized and solved in 30 μl of water. The rRNA was enzymatically hydrolyzed to ribonucleosides by 20 U benzonase (Santa Cruz Biotech, TX, USA) and 0.2 U nuclease P1 (Sigma Aldrich) in 10 mM ammonium acetate pH 6.0 and 1 mM magnesium chloride at 40 °C for 1h, then added ammonium bicarbonate to 50 mM, 0.002 U phoshodiesterase I and 0.1 U alkaline phosphatase (Sigma Aldrich) and incubated further at 37 °C for 1h. The hydrolysates were added 3 volumes of acetonitrile and centrifuged (16,000 g, 30 min, 4 °C). The supernatants were lyophilized and dissolved in 50 µl water for LC-MS/MS analysis of modified and canonical ribonucleosides. Chromatographic separation was performed using an Agilent 1290 Infinity II UHPLC system with an ZORBAX RRHD Eclipse Plus C18 150 x 2.1 mm ID (1.8 μm) column protected with an ZORBAX RRHD Eclipse Plus C18 5 x 2.1 mm ID (1.8 µm) guard column (Agilent Technologies). The mobile phase consisted of water and methanol (both added 0.1% formic acid) run at 0.23 ml/min, for modifications starting with 5% methanol for 0.5 min followed by a 2.5 min gradient of 5-15% methanol, a 3 min gradient of 15-95% methanol and 4 min re-equilibration with 5% methanol. A portion of each sample was diluted for the analysis of unmodified ribonucleosides which was chromatographed isocratically with 20% methanol. Mass spectrometric detection was performed using an Agilent 6495 Triple Quadrupole system with electrospray ionization, monitoring the mass transitions 268.1-136.1 (A), 284.1-152.1 (G), 244.1-112.1 (C), 245.1-113.1 (U), 282.1-150.1 (m^6^A and m^1^A), 282.1-136.1 (Am), 258.1-126.1 (m^5^C), 286.1-154.1 (ac^4^C), 298.1-166.1 (m^7^G and m^2^G), 296.1-164.1 (m^6,6^A), 259.1-127.1 (m^3^U), 258.1-112-1 (Cm), 298.1-152.1 (Gm), 259.1-113.1 (Um), and 245.1-155.1 (Y) in positive ionization mode.

### 2.8 Statistics

Gene expression and cytokine/chemokine secretion data were analyzed by linear mixed effects models using the lmer function in the lme4 package for R 4.0.3. For analysis of gene expression, observations, where the standardized residual was larger than 3 in absolute values, were considered outliers and excluded from the analysis. Nested random effects were included for treatment (i.e., nanomaterial), concentration and experiment number, (concentration was nested in treatment, and treatment was nested in experiment number). For statistical analysis of cytokine secretion, treatment and experiment number were combined in to one variable and then included as a random effect. For assessment of the combined inflammatory potential, random effects were included for exposure (i.e., treatment and experiment number combined) and protein level, with protein level nested in exposure. p-values were adjusted with the Benjamini & Hochberg (BH) step-up FDR-controlling procedure. Cell viability data was analyzed by one-way ANOVA and Dunnett’s test. p-values <0.05 were considered significant. Venn diagrams were created using http://bioinformatics.psb.ugent.be/webtools/Venn/. If not stated otherwise, graphs were created using GraphPad Prism 9.4.1 and multipaneled figures were created in GIMP 2.10.4.

## 3 Results

### 3.1 Characterization of nanomaterials

**Figure 1:**
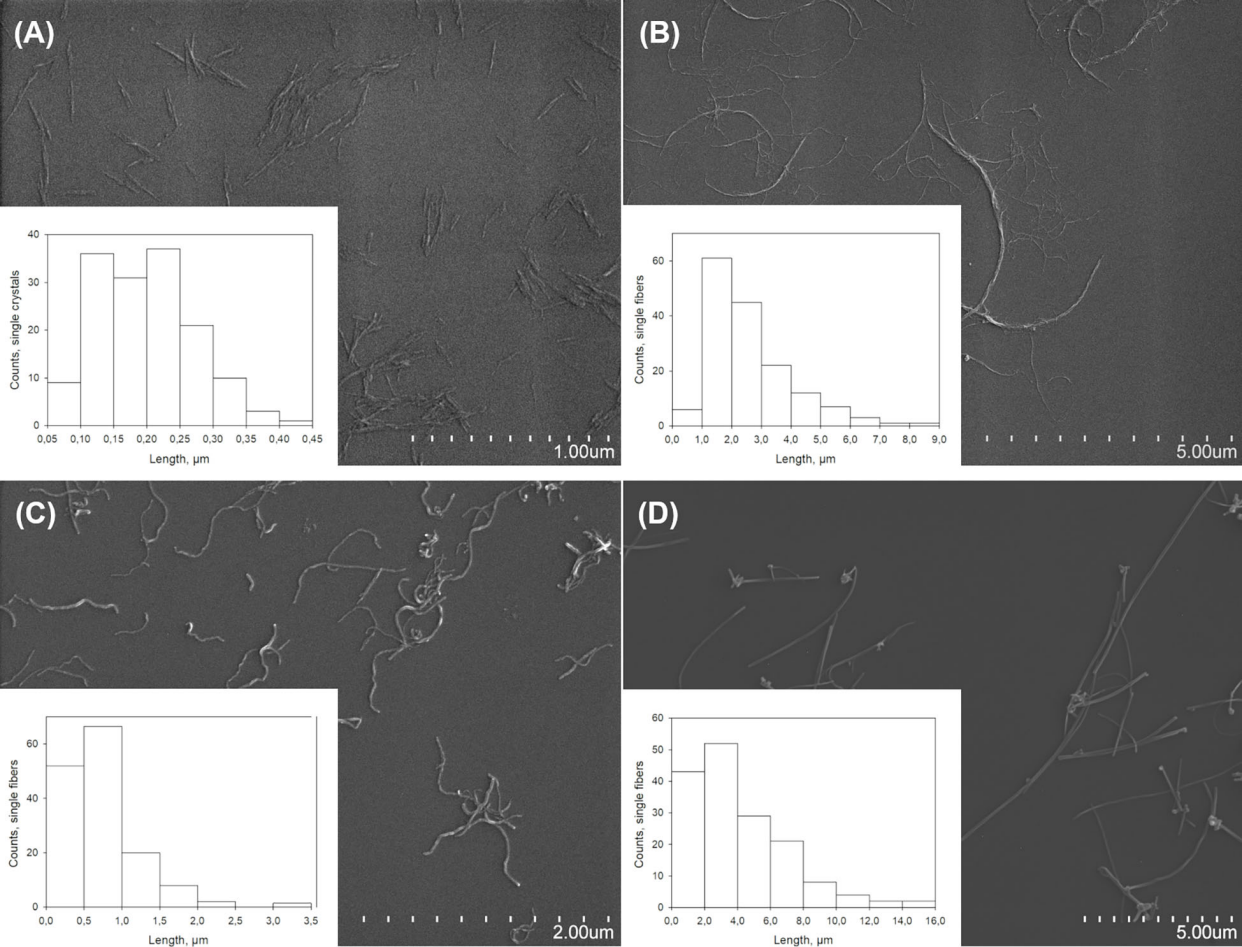
Characterization of nanomaterials. Representative SEM images and length measurements in µm of **(A)** CNC, **(B)** CNF, **(C)** NM-400, and **(D)** NM-401.

Physicochemical characterization of NC and MWCNT materials is presented in **Figure 1**. Size distribution of nebulized nanomaterials was determined by SEM analysis, with averages of 202 ± 73 nm in length and 15 ± 3 nm in width for CNC, **Figure 1A**, and long fibers of 2.63 ± 1.39 µm in length and 20 ± 10 nm in width for CNF, **Figure 1B**. Nebulized NM-400 fibers had an average length of 0.77 ± 0.50 µm and a width of 18 ± 4 nm, **Figure 1C**, and NM-401 had longer fibers of an average length of 4.10 ± 2.90 µm and a width of 93 ± 26 nm, **Figure 1D**. DLS measurements showed that CNC was well dispersed and had a hydrodynamic diameter of 119 ± 1 nm (polydispersity index: 0.14). The materials had calculated aspect ratios of 13.5 (CNC), 131.5 (CNF), 42.8 (NM-400), and 44.1 (NM-401), indicating that the fibrous particles included in this study are high aspect ratio nanomaterials.

### 3.2 Cellular uptake and effects on cell viability

**Figure 2:**
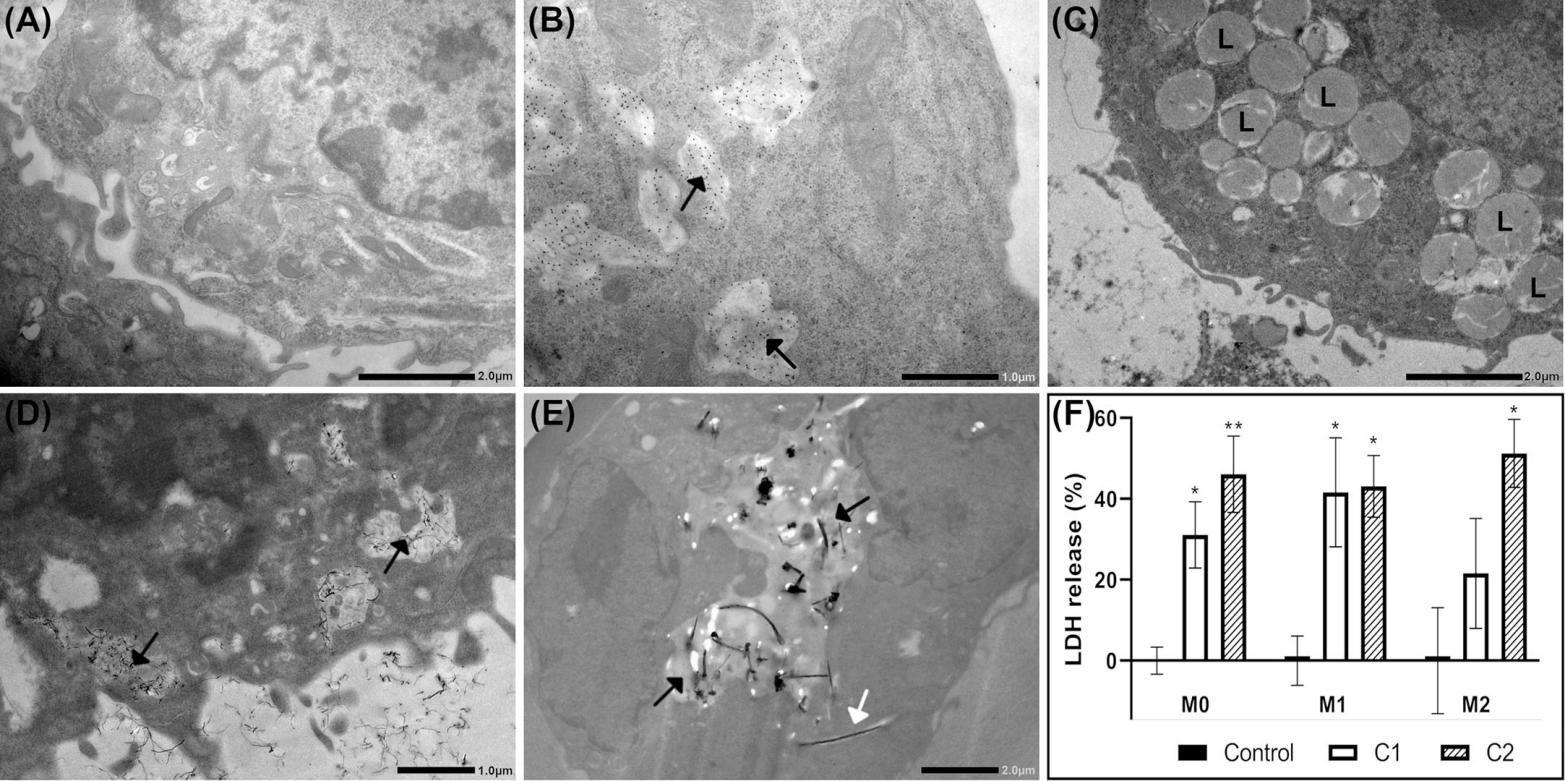
Cellular uptake and effects on membrane permeability at 24h post-exposure. Uptake of nanomaterials was investigated by TEM or immuno-TEM in M1 macrophages. Representative images of **(A)** Control, **(B)** CNC, **(C)** CNF, **(D)** NM-400, and **(E)** NM-401-exposed cells. C1: 0.15 μg/cm^2^ and C2: 2.7 μg/cm^2^. The experiment was repeated twice. Black arrows indicate endosomal structures with nanomaterials. White arrows indicate fibers in cell cytoplasm. L indicates lysosomal structures. **(F)** Membrane leakage as measured by medium lactate dehydrogenase (LDH) release following NM-401 exposure. Data indicate mean ± SD, (n=3-5), *p<0.05, **p<0.01.

Cellular uptake of nanomaterials was investigated by TEM or immuno-TEM. Cellular uptake 24h post-exposure was not affected by polarization status, **Supplementary Figure 2**. Representative images of cellular uptake of nanomaterials in M1 macrophages are shown in **Figure 2**. M1 macrophages exposed to dispersion media only (control cells), **Figure 2A**. CNC was highly taken up by all three macrophage phenotypes and was found predominantly within endosomes, as exemplified in M1 cells, **Figure 2B**. It should be noted that the uptake of nanocellulose materials were identified using immuno-TEM. Thus, the signal emanates from the gold labelled antibody used to detect the EXG-CNC complex and does not give any information to the size or shape of the particles taken up. CNF particles were not observed within exposed cells, **Figure 2C**. However, while CNF was not taken up, exposed cells had a high prevalence of lysosomal structures in the cytoplasm compared to controls, **Figure 2C**. In NM-400-exposed cells, fibers were found both within endosomes and in the extracellular space between adjacent cells, **Figure 2D**. NM-401-exposed cells showed fibers within endosomal structures but also partially in the cytoplasm, **Figure 2E**. Acridine orange staining, showed that nanomaterial exposure did not induce cytotoxicity at the investigated doses, **Supplementary Figure 3**. Altogether, these data show that CNC, NM-400 and NM-401 particles were taken up, whereas CNF particles were not phagocytosed by MH-S macrophages. Moreover, cells exposed to NM-401 had an increased dose-dependent leakage of LDH to the medium after 24h of exposure suggesting that NM-401 fibers may penetrate the cell membrane leading to LDH leakage, **Figure 2F**. This increase was not evident after 4h of NM-401 exposure nor in cells exposed to NC and NM-400, data not shown.

### 3.3 Effects of nanomaterial exposure on macrophage polarization markers

**Figure 3:**
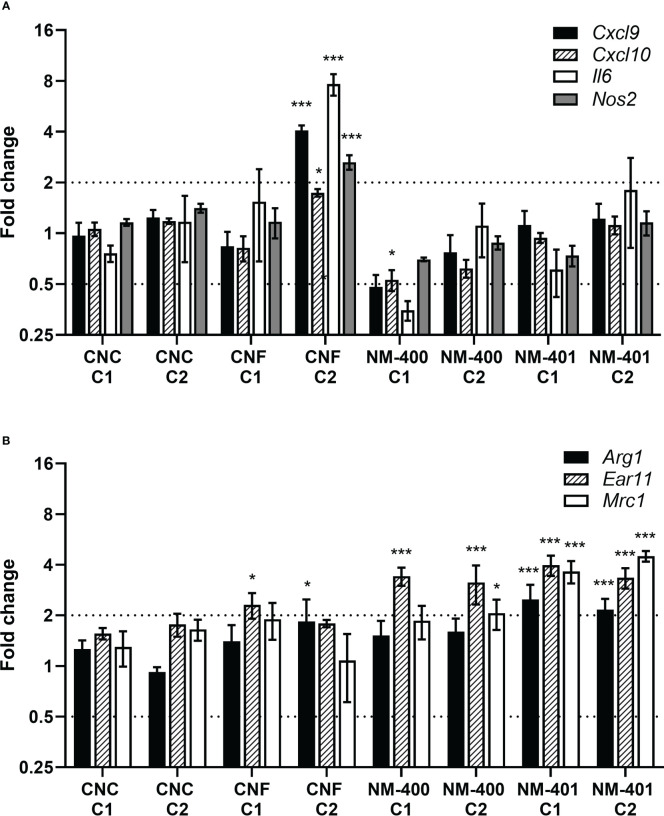
Effects of nanomaterial exposure on the expression of common macrophage polarization markers. Changes in gene expression were assessed by qPCR following exposure to CNC, CNF, NM-400 and NM-401 in **(A)** M1 and **(B)** M2 macrophages. C1: 0.15 μg/cm^2^ and C2: 2.7 μg/cm^2^. Expression was related to the mean expression in unexposed control cells which was set to 1. Data represent mean ± SE, (n=5), *p<0.05, ***p<0.001.

Effects of nanomaterial exposure on macrophage phenotype were assessed based on the expression of classical M1 and M2 markers. CNF exposure led to an enhanced M1 polarization with increased expression of *Cxcl9, Cxcl10, Il6* and *Nos2* in M1 cells, **Figure 3A**. On contrary, MWCNT exposure led to an increase in M2 markers. Both NM-400 and NM-401 induced the expression of the *Ear11* independent of dose, **Figure 3B**. Furthermore, NM-401 increased the expression of *Arg1* and *Mrc1* independent of dose, while NM-400 increased *Mrc1* expression only at the high dose (C2) in M2 macrophages, **Figure 3B**. Similarly, NM-400 (C1) treatment gave a 2.3-fold increase in *Mrc1* expression as well as a 0.4-fold decrease in *Cxcl10* expression in unpolarized M0 cells (p=0.010 and p=0.012, respectively), **Supplementary Table 2**. Exposure with CNC at the assessed doses did not affect the expression of macrophage polarization markers, **Figures 3A, B**. These data indicate that CNF induces the expression of common M1 markers, whereas NM-400 and NM-401 induce the expression of M2 macrophage markers at the tested doses.

### 3.4 Effects of nanomaterial exposure on cytokine and chemokine levels

**Figure 4:**
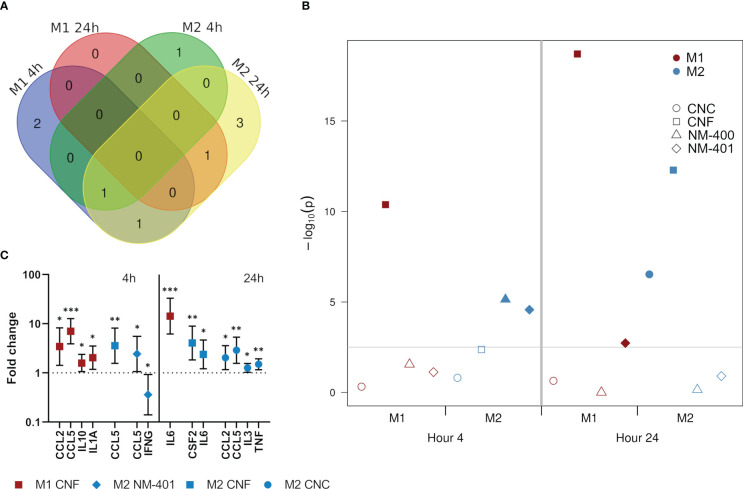
Effects on the secretion of cytokines and chemokines following exposure to CNC, CNF, NM-400 and NM-401 (high C2 dose). **(A)** Venn diagram illustrates the temporal differences in affected cytokines and chemokines in exposed M1 and M2 cells. **(B)** The combined inflammatory potential of nanomaterial exposure was illustrated by the p−values (−log_10_) of treatment effects for the deregulated proteins. The cutoff line indicates the significance level corrected for multiple testing. Filled symbols indicate values above the cutoff line where the exposure significantly affected the total secretion of cytokines in the specified macrophage subclass. **(C)** Cytokines and chemokines with significant changes in their secretion in M1 cells and M2 cells at 4 and 24h of exposure compared to controls. Data indicate mean ± CI, (n=4), *p<0.05, **p<0.01, ***p<0.001.

Secretion of a panel of predominantly pro-inflammatory cytokines and chemokines were quantified after 4h and 24h of nanomaterial exposure at the high dose (C2). Nanomaterial exposure led to an induction in the secretion of several cytokines and chemokines. This effect was especially prominent for M0 but also M1 cells, which both had similar response patterns at 4h, **Supplementary Figure 4**. Furthermore, NC materials induced stronger effects than MWCNT. At 4h, NC exposure induced the secretion of CCL2, CCL5, IL10 and IL1A independent of polarization phenotypes, while IL5, IL12p70 and TNF secretion was induced only in M0 cells. Interestingly, in M1 cells, the largest effects of exposure was observed at 4h, while in M2 cells the number of proteins affected were higher at 24h, **Figure 4A**. Analysis of the effects of treatment on the total cytokine release rather than on each individual cytokine separately (used as an estimation of inflammatory potential) showed that CNF exposure had the most pronounced overall effect on cytokine release in M1 macrophages at both 4 and 24h of exposure, whereas responses in M2 cells by both CNF and CNC exposure was delayed and evident only after 24h, **Figure 4B**. In M1 cells the largest effect of CNF exposure was observed in CCL2 (3.4-fold, p=0.017) and CCL5 (7.0-fold, p<0.001) at 4h, and in IL6 (14.3-fold, p<0.001) at 24h, **Figure 4C**. While MWCNT gave overall lower changes in the measured cytokine levels, an early response to MWCNT were observed in M2 cells, **Figure 4B**, where CCL5 levels were induced (2.4-fold, p=0.049) and IFNG levels reduced (0.4-fold, p=0.047) following 4h of NM-401 exposure, **Figure 4C**. Notably, CCL5 secretion was uniformly increased in both phenotypes and by both NC and MWCNT materials.

### 3.5 Epigenetic regulation of nanomaterial-modulated macrophage polarization

#### 3.5.1 Genes regulating epigenetic modifications

**Figure 5:**
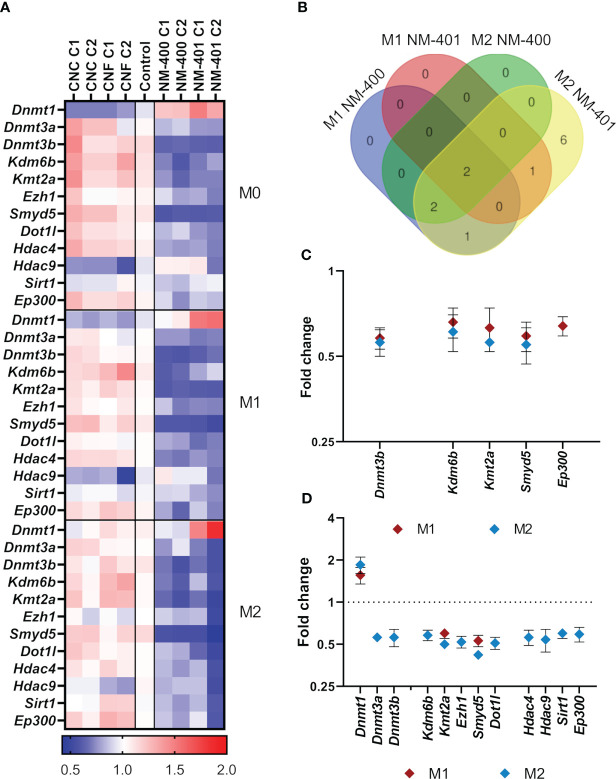
Effects on the expression of genes regulating epigenetic modifications. Changes in gene expression were assessed by qPCR following exposure to CNC, CNF, NM-400 and NM-401. C1: 0.15 μg/cm^2^ and C2: 2.7 μg/cm^2^. **(A)** Heatmap of the mean fold changes in regulated genes following 24h of nanomaterial exposure in M0, M1 and M2 macrophages. **(B)** Venn diagram illustrates commonly regulated genes in M1 and M2 cells after NM-400 and NM-401 exposure. **(C)** Genes significantly regulated following exposure to NM-400. **(D)** Genes significantly regulated following exposure to NM-401. Data represent mean ± SD, (n=5), p<0.05.

The expression of genes regulating histone methylation (*Prt1*, *Smyd2*, *Smyd3*, *Smyd5*, *Kmt2a*, *Ezh1*, *Ezh2*, *Suv39h2*, *Dot1l*, *Wdr5*, *Ash1l*, *Setd7*, *Kdm1a*, and *Kdm6b*), histone acetylation (*Hdac2*, *Hdac3*, *Hdac4*, *Hdac9*, *Sirt1*, *Sirt2*, *Kat3a*, *Kat3b/Ep300*, *Kat5*, *Kat6a*, and *Kat6b*), and DNA methylation (*Dnmt1*, *Dnmt3a*, and *Dnmt3b*), was assessed in M0, M1 and M2 macrophages exposed to nanomaterials for 24h, **Figure 5A**, **Supplementary Table 3**. MWCNT exposure resulted in more alterations in the analyzed markers compared to NC, which only showed trends to an increase in *Kdm6b* and a reduction in *Hdac9* expression, **Figure 5A**. MWCNT exposure reduced the expression of several genes regulating DNA methylation and histone modifications. Although the overall effects of nanomaterial exposure on the expression of epigenetic regulators were moderate, the most prominent effect was observed in M2 cells exposed to NM-401 high dose (C2), where 12 genes were found to be differentially regulated, **Figure 5B**. Of these, 7 genes (*Dnmt1*, *Dnmt3a*, *Ezh1*, *Dot1l*, *Hdac4*, *Hdac9*, and *Sirt1*) were exclusively regulated by NM-401, whereas 5 genes (*Dnmt3b*, *Kdm6b*, *Kmt2a*, *Smyd5*, and *Ep300*) were regulated by both MWCNT materials, **Figures 5C, D**. Only *Kmt2a* and *Smyd5* were regulated by both MWCNT in both cell types, **Figures 5C, D**. Exposure at the low dose resulted in similar trend in effect as the high doses for each nanomaterial, **Figure 5A**, **Supplementary Table 3**.

#### 3.5.2 miRNAs

**Figure 6:**
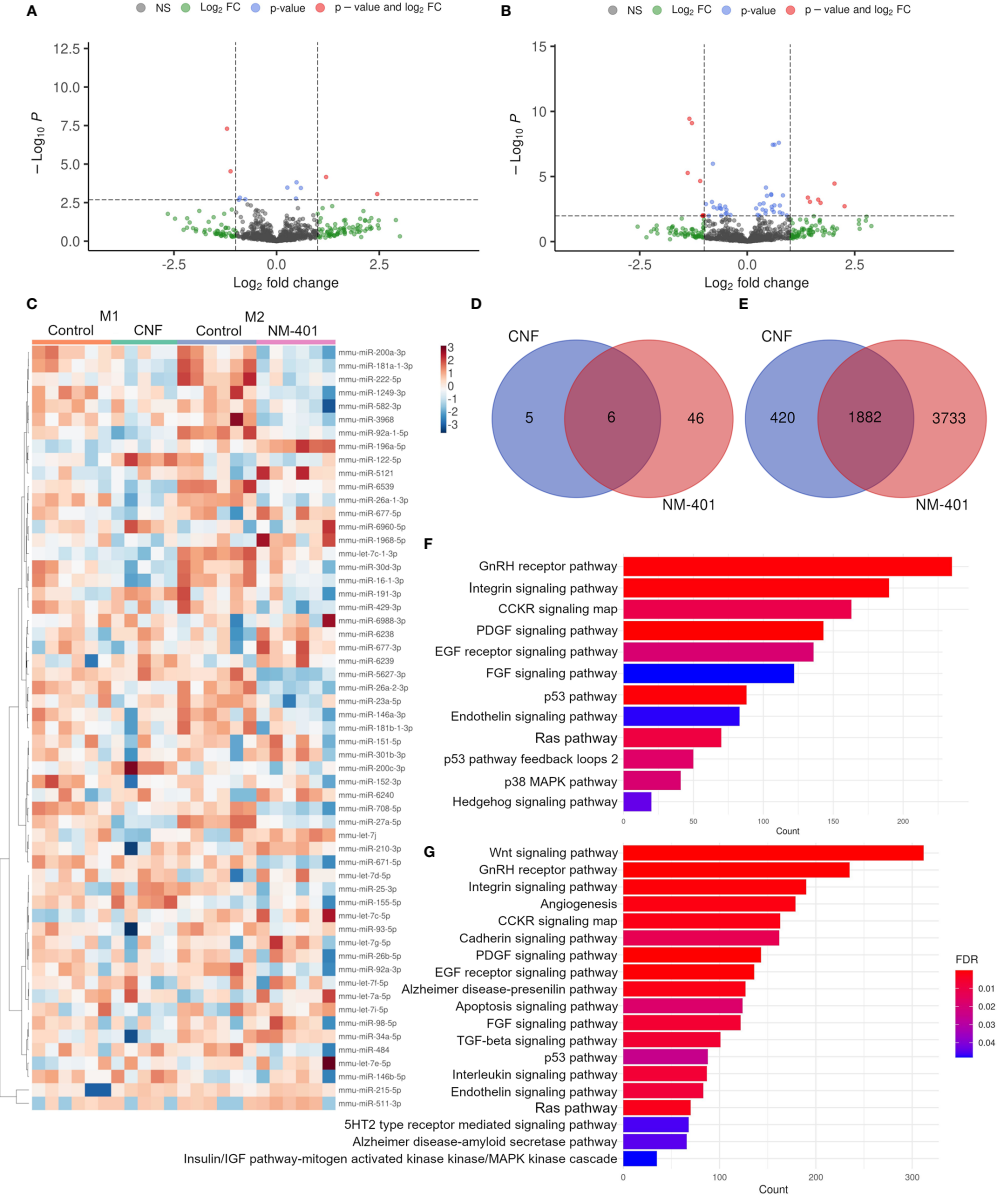
CNF and NM-401 exposure induced changes in miRNA expression. **(A)** Volcano plot of differentially expressed known miRNAs in CNF-exposed M1 cells. **(B)** Volcano plot of differentially expressed known miRNAs in NM-401-exposed M2 cells. Y-axes show negative decadic logarithm of uncorrected p-values (-log10 P), x-axes show the binary logarithm of fold changes (log2 fold change). Log2 fold change cutoff = 1, and FDR cutoff = 0.1 are indicated. **(C)** Clustering heat map of differentially expressed miRNAs -log_10_(CPM). **(D)** Venn diagrams of differentially expressed miRNAs and **(E)** their predicted target genes. PANTHER analyzes of the target genes of differentially expressed miRNA in **(F)** CNF- and **(G)** NM-401-exposed macrophages, (n=5-6).

The involvement of miRNA regulation in CNF and NM-401-induced macrophage polarization was assessed by miRNA NGS. CNF and NM-401 were selected based on their observed ability to enhance M1 polarization (CNF) and M2 polarization (NM-401). Nanomaterial exposure altered the expression (FDR < 0.1) of 11 and 52 miRNA in CNF-exposed M1 macrophages and NM-401-exposed M2 macrophages, respectively. Of these, 4 miRNAs were significantly regulated in CNF-exposed M1 cells and 11 miRNAs were significantly regulated in NM-401 exposed M2 cells, (FDR < 0.1, p<0.05), **Figures 6A, B**. **Figure 6C** shows heatmap and clustering analysis of the identified miRNAs. Moreover, six miRNAs (miR-26a-2-3p, miR-26a-1-3p, miR16-1-3p, miR155-5p, miR-27a-5p and miR-25-3p) were regulated by both nanomaterials indicating that these miRNAs may be common regulators of macrophage phenotypic alterations following nanofiber exposure, **Figure 6D**. CNF led to a >2-fold increase in the expression of miR-122-5p and >2-fold reduction in miR-16-1-3p and miR-27a-5p expression in M1 macrophages. Of the 52 miRNAs regulated following NM-401 exposure, miR-511-3p, miR-677-3p, miR-5121 and the unverified miRNAs miR-6238, miR-6239 and miR-6240 were >2-fold upregulated whereas let-7c-1-3p, miR-708-5p, miR-26a-2-3p and miR27a-5p were >2-fold downregulated. Fold changes and adjusted p-values are available in **Supplementary Data Sheet 1**. Predicted mRNA targets for the differentially expressed miRNAs, **Figure 6E**, **Supplementary Data Sheet 2**, were used for statistical overrepresentation test in PANTHER Pathways. Pathway analysis showed that targets of the regulated miRNAs were enriched in growth factor (PDGF, EGF and FGF), RAS/MAPK, CCKR, GNRHR, integrin, and endothelin signaling pathways, **Figures 6F, G**. These pathways are important in inflammation or in the activation, polarization, migration, and regulation of the phagocytic capacity of macrophages. In addition, pathways involved in interleukin, WNT and TGFB signaling were highly enriched for the NM-401 differentially expressed miRNAs, **Figure 6G**.

#### 3.5.3 rRNA modifications

To assess the potential involvement of epitranscriptomic regulation on nanomaterial-induced macrophage polarization, posttranscriptional base modifications of rRNA were assessed. CNF and NM-401 were selected based on their observed ability to enhance M1 polarization (CNF) and M2 polarization (NM-401). The confirmed rRNA modifications (50): m^1^A, m^6^A, m^6,6^A, m^5^C, ac^4^C, m^7^G, m^2^G, Y, m^3^U, and the 2’-O-Me (Am, Cm, Gm, and Um) were analyzed. Exposure to CNF and NM-401 did not induce changes in the analyzed rRNA modifications, **Supplementary Table 4**.

## 4 Discussion

Inhalation of high aspect ratio nanomaterials evokes inflammatory responses in the lung, which if left unresolved may evolve into pulmonary fibrosis and cancer (18, 29, 51–54). It is well documented that the inhalation of persistent NC leads to acute pulmonary inflammation which is gradually time-dependently alleviated and does not result in fibrosis (15, 17–20, 28). In accordance with previous studies (23, 26, 27), we here show that NC materials trigger proinflammatory responses and enhance macrophage M1 phenotype. CNF alone did not alter the expression of the analyzed macrophage polarization markers in M0 cells, however induced the secretion of the proinflammatory cytokines IL5, IL12 and TNF. Furthermore, CNF exposure enhanced the expression of the M1 markers *Cxcl9*, *Cxcl10*, *Il6*, and *Nos2* in IFNG-activated M1 macrophages, and resulted in a marked increased secretion of e.g., CCL5 and IL6 after 4h exposure. Thus, CNF exposure highly induced the expression of IL6 both on mRNA and protein level. Similarly, NC exposure enhances both CXCL9 mRNA and protein expression (27). CNC did not affect the expression of macrophage polarization markers at the doses investigated. We have previously shown that CNC enhanced M1 phenotype and trigged secretion of proinflammatory cytokines at higher doses (15 ug/cm^2^) (27). Interestingly, CNC materials typically induce more pronounced inflammation than CNF materials in exposed animals. Concurrently, CNC is rapidly internalized by macrophages and found within endosomes in the cell cytoplasm at 24h of exposure. We have previously demonstrated that CNC particles are taken up through phagocytosis/micropinocytosis *via* actin and PI3K-dependent mechanisms already after 2h of exposure (27). CNF on contrary, is not effectively internalized by alveolar macrophages *in vitro* as demonstrated here by immuno-TEM analysis, however exposed cells showed an increased presence of lysosomal structures. These findings are supported by previous studies showing increased presence of vacuoles in the cytoplasm of CNF exposed cells despite low or no particle uptake (24, 55). Previous studies also suggest that CNF may absorb to the plasma membrane resulting in limited uptake and that its effects may involve receptor-mediated mechanisms (23, 56, 57). Thus, these findings indicate that the pulmonary inflammation induced by CNC and CNF materials may involve different cellular mechanisms. Furthermore, the different shape and physical characteristics of these two materials likely influence their effects and the fiber shape of CNF may contribute to the stronger pro-inflammatory effects observed. Indeed, it is well acknowledged that fibers and high aspect nanomaterials may induce prominent sustained inflammation upon inhalation and may even result in pulmonary fibrosis or cancer (58).

Despite the high aspect ratio of CNF materials, their toxic pulmonary responses differ from that caused by MWCNT and asbestos (18, 28). Contrary to NC, MWCNT, similarly to asbestos, are inefficient cleared and retained in the lung resulting in chronic inflammation, fibrotic lesions, lung cancer and mesothelioma in long-term exposed animals (51, 59–64). In addition to differences in chemical composition, CNF particles are also highly coiled and branched, affecting their uptake. It is generally acknowledged that high rigidity of particles highly influences their clearance and the physiological responses upon inhalation, as they may induce damage to endosomes and phagosomes or directly pierce the cells leading to prolonged and more prominent inflammatory responses (65–68). For MWCNT, a progression from acute inflammation to chronic fibrotic changes suggests that a resolution of inflammation involving Th2 responses may underlie the fibrotic events (38). It is also evident that long and rigid MWCNT typically induce more prominent inflammation and fibrotic responses than shorter coiled MWCNT (61, 64, 69). In agreement, this study showed that the long and rigid NM-401 fibers gave more severe effects on macrophage markers, as illustrated by increased M2 markers *Arg1*, *Ear11*, and *Mrc1*, and cytokine secretion, compared to the shorter and more coiled NM-400 fibers. As both materials have similar aspect ratios (NM-400: 42.8 and NM-401: 44.1), these differences could possibly to some extent be attributed to the rigidity of the materials. It is well described that rod-like CNT may induce more severe fibrotic responses (61, 64) and may disrupt macrophage function due to unsuccessful uptake resulting in frustrated phagocytoses and damage of surrounding tissues (58, 67). Analyzes of cellular uptake showed that although both NM-400 and NM-401 were internalized and found within endosomes in alveolar macrophages, NM-401 fibers were also found in the cytoplasm after 24h of exposure. Previous studies have demonstrated that MWCNT may be taken up by macrophages already after 1h of exposure (70). Further analyzes indicated that NM-401-exposed cells showed signs of membrane leakage at the sub-toxic concentrations tested. This may suggest that the more rigid NM-401 fibers induce cellular membrane damage which may contribute to the enhanced effects observed by these fibers. In agreement with our findings, a grouping effort of MWCNT for risk assessment (69) conclude that NM-400 occurs as tangled agglomerates which are not able to induce frustrated phagocytosis (65), while NM-401 cause lysosomal disruption, intracellular ‘vesicle escape’ (66) and frustrated phagocytosis in macrophages both *in vitro* and *in vivo* (65). As several studies have demonstrated that MWCNT-induced fibrosis is Th2-type response mediated (reviewed in 38), the involvement of M2 polarization events has been suggested (35–37). Our data confirm an enhanced M2 phenotype following MWCNT exposure, which is more prominent for long rigid fibers. This further supports the involvement of M2 macrophage phenotype in the onset of MWCNT-induced lung fibrosis.

The involvement of epigenetic events in the observed fine-tuning of macrophage phenotype in response to nanofiber exposure has not been clarified. Here we demonstrate that the enhanced M1 phenotype observed after CNF exposure did not involve histone or DNA modification events. However, the MWCNT-induced M2 phenotype showed regulation of several histone and DNA modifying enzymes which may be of importance in fibrosis onset following MWCNT exposure. The epigenetic regulation of M1 macrophage phenotypes has been extensively studied and involves KDM6B (previously known as JMJD3), KMT6, several HDACs and DNMT1 (6). However, fever studies have focused on the role of histone and DNA modifying enzymes in M2 macrophage polarization (71–74). In murine macrophages, alternative M2 phenotype is mediated by the histone H3K27 demethylase *Kdm6b* which may regulate the expression of e.g., *Irf4*, *Chi3l3*, *Retn1a*, and *Arg1* (71, 74). Furthermore, *Kdm6a* deficient mice show enhanced M2 macrophage polarization (75). These findings indicate that the KDM6 family is important in regulating the M2 phenotype. Interestingly, in this study, both MWCNT decreased the expression of *Kdm6b* in M2 cells, supporting a potential role of H3K27 methylation in the regulation of macrophage phenotype associated with increased expression of *Arg1*, *Mrc1*, and *Ear11* following MWCNT exposure. Furthermore, the expression of the H3K4 methyltransferases *Kmt2a* (previously known as *Mll*) and *Smyd5* mRNA was reduced by MWCNT in both M1 and M2 macrophages, while the *Ezh1* and *Dot1l* mRNA were downregulated exclusively by NM-401 in M2 macrophages. The HMT family members hold diverse roles in macrophage polarization as they regulate the expression of inflammatory genes. In general, HMTs promote M2 phenotype by repressing the expression of pro-inflammatory cytokines e.g., *Tnf*, *Il1b*, *Il6*, and *Cxcl10* (76, 77). On the other hand, KMT2A is required for M1 macrophage polarization as it leads to enhanced *Cxcl10* expression (72). In addition to alterations in HMT expression, we also observed a reduction in the expression of *Hdac4*, *Hdac9* and *Sirt1* as well as the HAT *Kat3b (Ep300)* in M2 cells following NM-401 exposure. HDACs are recognized as important regulators of polarization as their inhibition results in altered levels of cytokines, chemokines and macrophage activation markers (5, 78). Accordingly, *Hdac9* deficiency has been shown to exaggerate M2 macrophage polarization in mouse and human macrophages by upregulating M2 markers e.g., *Mrc1* (also known as *Cd206*) and *Pparg* and repressing markers involved in M1 polarization e.g., *TNF*, *IL6* and *CXCL10* (79, 80). Similarly, HDAC4 inhibits NFKB signaling and increase *Tnf* and *Il6* expression in M1 cells (81). On contrary, SIRT1 represses M1 phenotype by reducing the expression of IKK/NFKB and JNK regulated inflammatory target genes e.g., *Tnf, Il1b, Il6, Il12, Nos2*, and *Mcp1* (82, 83).

While histone modifications are important in the regulation of macrophage polarization and their involvement in nanomaterial-induced pulmonary effects is evident (84, 85), the role of histone modifications during macrophage phenotypic changes in response to nanomaterial exposure has not been previously explored. Thus, while it is difficult to infer the direct effects the epigenetic regulatory enzymes have on the genetic profiles of activated macrophages it is evident that they are important in the fine-tuning of macrophages’ responses to MWCNT exposure. For the MWCNT-enhanced changes in macrophage phenotype our data emphasize the role of the HMTs *Kmt2a* and *Smyd5* as their expression is universally downregulated by both MWCNT and shows the largest effect of all modifying enzymes investigated in this study. In support of our data, it is well known that KMT2A is essential for M1 polarization and its downregulation by MWCNT may be important for the triggering of an enhanced M2 phenotype. SMYD5 is a relatively unknown member of the SMYD family proteins. To date, there is only one study proposing a role of SMYD5 and H4K20me3 in the repression of TLR4 target genes in macrophages (76). However, our data indicate that SMYD5 may also have a role in the regulation of the alternative M2 phenotype. Thus, the downregulation of HMTs observed in our study suggests that their activity is important in MWCNT-induced macrophage polarization and that methylation of H3K4, H3K27 and H3K79 may be critical in modifying macrophage phenotypes in response to nanomaterial exposure. Furthermore, our data support a role of HDACs in nanomaterial-induced inflammation and more specifically our findings indicate that histone acetylation events regulated by HDAC4, HDAC9 and SIRT1 may be critical in the Th2 responses to MWCNT.

The interplay between histone modifications and other epigenetic mechanisms such as nucleotide modifications may add further complexity and may be critical for macrophages’ ability to adjust and reprogram to changes in their environment. It has been shown that MWCNT induce changes in DNA methylation in the lungs of exposed animals (86–88), as well as in MWCNT-exposed workers (89). Furthermore, NM-400-exposure of monocytes induces DNA hypomethylation of inflammation-related genes and genes involved in macrophage polarization, e.g., the JAK-STAT pathway (90). In this study changes in DNA methylation were not directly measured, however, a deregulation of the expression of DNA methyltransferases was observed following MWCNT exposure, where MWCNT induced *Dnmt1* and reduced *Dnmt3a* and *Dnmt3b* expression in M2 macrophages. DNMT1 and DNMT3B are important in the regulation of macrophage polarization as their overexpression usually results in induced expression of proinflammatory cytokines and concurrent M1 polarization, whereas their depletion leads to enhanced M2 macrophage polarization (12–14). Altogether, these finding support a potential role of DNA methylation events in MWCNT induced M2 polarization.

A well-recognized level of epigenetic regulation is exerted by non-coding RNAs, which affect the expression of various genes involved in macrophage polarization. In this study, both CNF and NM-401 exposure induced changes in miRNA transcript levels. The target genes of the differentially expressed miRNA were involved in several different signaling pathways, e.g., growth factor, Ras/MAPK, CCKR, GnRH-R, and integrin signaling, which are important for inflammation and for the activation, polarization, and function of macrophages. More specifically, CNF exposure resulted in a 2-fold upregulation of miR-122-5p and downregulation of miR-16-1-3p, and miR-27a-5p expression in M1 cells. Recent studies suggest a role of miR-122-5p in pulmonary inflammation and in the regulation of pro-inflammatory cytokine expression e.g., TNF, IL1B, IL6, and MCP1 (91, 92). Furthermore, miR-122-5p may induce M1 polarization (93). While the role of miR-16-1-3p in macrophage polarization has not been investigated, it is suggested to modulate the IL6-JAK-STAT3 signaling pathway (94). On contrary, more targets are described for miR-16-5p which has been suggested as a promotor of M2 polarization (95). These finding are consistent with our data showing that miR-122-5p is highly upregulated and miR-16-1-3p downregulated following CNF exposure, which is associated with increased secretion of IL6, CCL2, and CCL5 and an enhanced M1 phenotype. Interestingly, a 0.4-fold change in miR-27a-5p expression levels was observed for both CNF and NM-401. The role of miR-27a-5p in macrophage polarization is not well understood, as different studies have demonstrated induced expression of miR-27a-5p in both M1 and M2 polarization (96, 97). Furthermore, miR-27a is suggested to suppress PPARG signaling which is involved in the control of inflammatory responses by repressing pro-inflammatory signaling pathways such as JUN (previously known as AP-1), NFKB and STAT3, consequently enhancing M1 polarization (98). Thus, while it is evident that this miRNA is important in macrophage polarization more studies are needed to understand the exact regulation it exerts. Moreover, while the role of miR27-a has not been previously demonstrated in nanofiber-induced macrophage polarization, TiO_2_ exposure decreases the expression of miR-27a-5p in murine macrophages (99). Furthermore, miR-155-5p and miR-25-3p were also regulated by both CNF and NM-401 exposure in this study. Especially, miR-155 is recognized as a major regulator of inflammation and macrophage polarization and has several known target mRNAs involved in cytokine signaling. miR-155 alters macrophage phenotype through various signaling pathways including the STAT6 and JNK pathways (100–102). In addition, CEBPB is a direct target of miR-155 (103). MWCNT exposure has previously been shown to reduce the expression of miR-155-5p in BEAS-2B cells (104). Moreover, both polystyrene and TiO_2_ nanomaterials reduce the expression of miR-155-5p in THP1 monocytes (105). Together these data indicate that miR-27a-5p and miR-155-5p may be common regulators of macrophage phenotypes in response to various nanomaterial exposures.

NM-401 exposure also led to a prominent upregulation of miR-511-3p and miR-677-3p and a downregulation of miR-708-5p, miR-26a-2-3p and let-7c-1-3p. While very little information is available on the function of miR-677-3p, miR-708-5p, miR-26a-2-3p and let-7c-1-3p in macrophage polarization, they have been indicated roles in immune responses. Accordingly, miR-708 has been suggested as a suppressor of TNF/IL1B signaling leading to reduced IL6 levels in pulmonary cells (106). Furthermore, let-7c-1-3p is induced in M2 macrophages and both let-7c-1-3p and miR-26a-2-3p are involved in cytokine-cytokine receptor interactions (97). Accordingly, let-7c-1-3p targets immune response genes e.g., *Ccr9*, *Il15*, *Cxcl10* and *Ccl2*, whereas miR-26a-2-3p targets e.g., *Il15ra*, *Ccl7*, *Cx3cl1*, *Cxcl11* and *Il1b* (97). On contrary, miR-511-3p is acknowledged as regulator of M2 polarization. miR-511 is a putative positive regulator of Toll-like receptor 4 in macrophages and is involved in the Th cell polarization through modulation of *MRC1* expression (107, 108). Moreover, downregulation of miR-511-3p alters PPARG activity leading to downregulation of pro-inflammatory cytokine production in dendritic cells (109). In accordance, MRC1 has been shown to regulate macrophage polarization through miR-511-3p in mice. MRC1 depletion resulted in reduced miR-511-5p levels and enhanced M1 polarization whereas, enhanced miR-511-3p levels resulted in M2-driven anti-inflammatory responses (110). Furthermore, miR-511 has been shown to be highly expressed in IL4-stimulated (M2a) macrophages (111). Thus, while the contribution of miR-677-3p, miR-708-5p, miR-26a-2-3p and let-7c-1-3p on MWCNT-enhanced M2 polarization need further investigation, the increased miR-511-3p expression levels observed in this study could suggest a role of miR-511-3p in the enhanced M2 macrophage phenotype observed following MWCNT exposure.

Finally, RNA modifications have been recently suggested as an additional level of epigenetic regulation in response to various environmental stimuli. For instance, air pollution and PM2.5 exposure has been shown to affect the global N6-methyladenosine (m^6^A) and mRNA 5-methylcytidine (m^5^C) levels (112, 113). Moreover, RNA modifications, specifically m^6^A, have critical roles in immune cell function and immune responses, and have been implicated in various aspects of macrophage biology, including macrophage polarization (114–116). Considering this, RNA modifications could contribute to adjustment of macrophage responses to various nanomaterials. However, our data show that CNF and MWCNT did not induce any changes in the levels of known rRNA modifications.

Altogether, this study demonstrates that CNF exposure enhances M1 macrophage, while MWCNT exposure enhances M2 macrophage polarization, congruent with the observed effects of these materials in triggering inflammation and fibrosis, respectively, in exposed animals. These data support the importance of macrophage phenotypic changes in the onset and resolution of nanofiber-induced inflammation and fibrosis and emphasize the importance of epigenetic regulation in the fine-tuning of macrophages. In correspondence with its stronger immunogenic effects, the MWCNT-induced changes in macrophage polarization involved more prominent epigenetic regulatory events i.e., histone modifications, DNA methylation and miRNAs. Whilst, epigenetic modifications are often investigated separately, there is substantial cross talk between mechanisms to establish the epigenetic landscape. In light of this, our study provides important novel evidence illustrating the intricacy of the epigenetic regulation in macrophages in response to environmental changes. Further, identifying epigenetic patterns in macrophages which may be important in nanofiber-induced inflammation and fibrosis.

## Data availability statement

The datasets presented in this study can be found in online repositories. The names of the repository/repositories and accession number(s) can be found below: PRJNA902122 (SRA).

## Author contributions

Conceptualization: JE, DE, JT and SZ-N. Methodology: JE, TZ, TE, KA, AK and JC. Bioinformatics and statistics: TZ, ØS and TH. Data curation: JE, ØS and TH. Writing – original draft: JE, TZ and TE. Writing – reviewing and editing: JE, TZ, JC, JT and SZ-N. All authors contributed to the article and approved the submitted version.
