# Supplementary material for: High aspect ratio nanomaterial-induced macrophage polarization is mediated by changes in miRNA levels
Source: Front Immunol. 2023 Jan 27;14:1111123. doi: 10.3389/fimmu.2023.1111123 (PMC9911541; doi:10.3389/fimmu.2023.1111123)
Supplement: Supplementary file 1 [file Presentation_1.pdf]

Supplementary Material

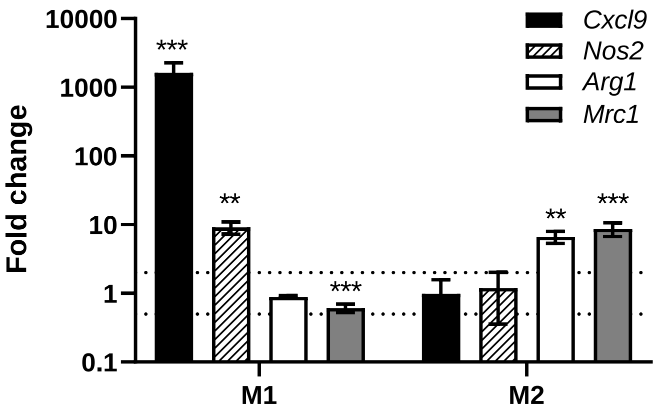

Supplementary Figure 1. Characterization of macrophage polarization. Air-lifted MH-S macrophages were polarized towards M1 or M2 phenotypes for 24h with IFNG and IL4/IL13, respectively. Gene expression of M1 markers (*Cxcl9* and *Nos2*) and M2 markers (*Arg1* and *Mrc1*) was assessed by qPCR. Reported values represent fold changes in gene expression relative to the mean expression in unstimulated macrophages (without addition of activators), which was set to 1. Data represent mean ± SD, (n=5), **p<0.01, ***p<0.001.

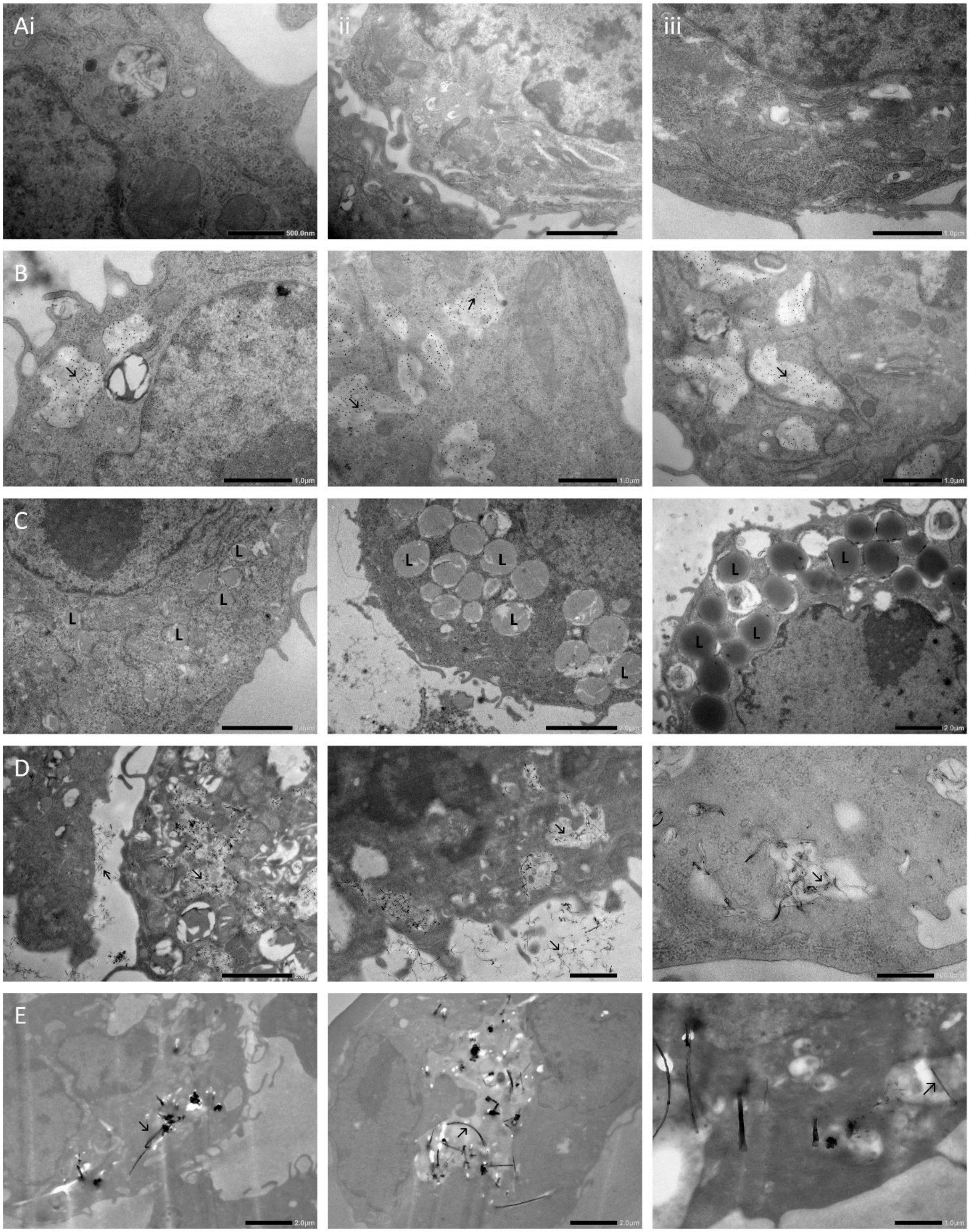

Supplementary Figure 2. Cellular uptake of nanomaterials. Representative TEM images show nanoparticle uptake in i) M0, ii) M1, and iii) M2 macrophages 24h after exposure with A) dispersion

media (control), B) CNC, C) CNF, D) NM-400, and E) NM-401. The experiment was repeated twice. Black arrows indicate endosomal structures with nanomaterials. White arrows indicate fibers in cell cytoplasm. L indicates lysosomal structures.

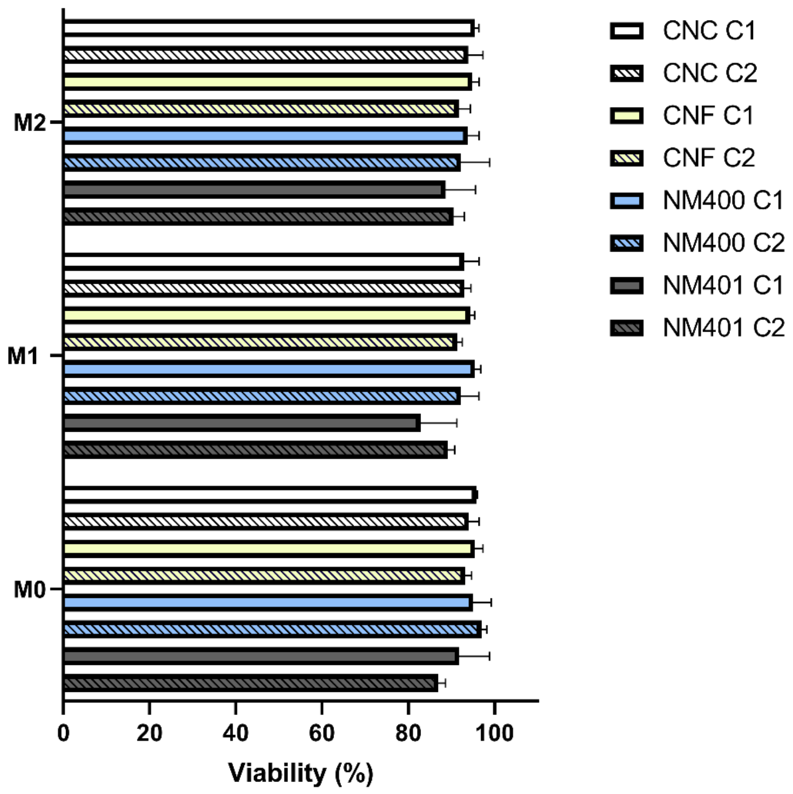

Supplementary Figure 3. Effects of nanoparticle exposure on cell viability. Cell viability was assessed in unpolarized (M0), M1 and M2 macrophages 24h after exposure to CNC, CNF, NM-400 and NM-401. C1: 0.15 µg/cm^2 and C2: 2.7 µg/cm^2. Data indicate mean±SEM, n=3.

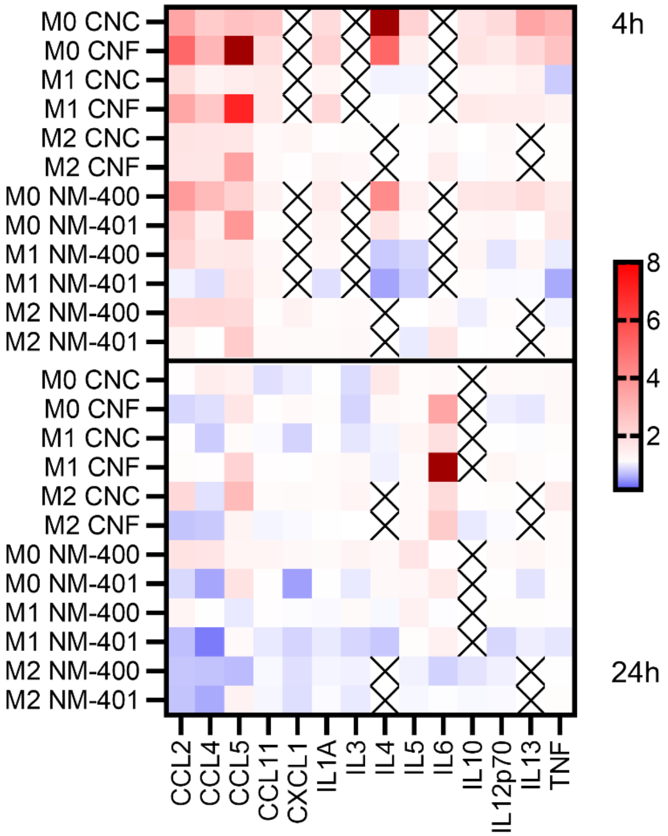

Supplementary Figure 4. Changes in secreted cytokine and chemokine levels following nanoparticle exposure to CNC, CNF, NM-400 and NM-401 (high C2 dose) in unpolarized (M0), M1 and M2 macrophages. Data indicate average fold change values of proteins expressed in more than one cell type. Fold changes >8 are indicated in dark red, (n=4).

Supplementary Table 1. Primer sequences.

Gene name	Forward	Reverse
Ubc	GAGACGATGCAGATCTTTG	ATGTTGTAGTCTGACAGGG
Hprt	AGGGATTTGAATCACGTTTG	TTTACTGGCAACATCAACAG
Cxcl9	GAGGAACCCTAGTGATAAGG	GTTTGATCTCCGTTCTTCAG
Il6	AAGAAATGATGGATGCTACC	GAGTTTCTGTATCTCTCTGAAG
Ear11	CAAAGCAGACTGGGAAAC	GGGGATAGGCTCTGTTATAG
Mrc1	ATATGAGCCAAATGATGAGC	CATCCTTGCCTTTCATAACC

Supplementary Table 2. Effects of nanoparticle exposure on the expression of classical M1 and M2 markers. C1: 0.15 µg/cm^2 and C2: 2.7 µg/cm^2. Significant fold changes (≥ 1.5 or ≤ 0.67, p<0.05) are indicated in bold, (n=5).

Gene symbol	Cell	Treatment	Fold changes (mean±SD)	Adj.p-value
Arg1	M0	CNC C1	1.03±0.23	0.9600
Arg1	M0	CNC C2	0.98±0.16	0.9400
Arg1	M0	CNF C1	1.24±0.23	0.5100
Arg1	M0	CNF C2	1.39±0.24	0.2400
Arg1	M0	NM-400 C1	0.97±0.26	0.9100
Arg1	M0	NM-400 C2	0.90±0.13	0.8300
Arg1	M0	NM-401 C1	1.27±0.15	0.5800
Arg1	M0	NM-401 C2	1.32±0.32	0.4600
Arg1	M1	CNC C1	0.89±0.09	0.7200
Arg1	M1	CNC C2	0.90±0.12	0.7600
Arg1	M1	CNF C1	1.11±0.12	0.8300
Arg1	M1	CNF C2	1.10±0.10	0.8600
Arg1	M1	NM-400 C1	0.77±0.17	0.5800
Arg1	M1	NM-400 C2	0.78±0.25	0.5100
Arg1	M1	NM-401 C1	1.04±0.14	0.9400
Arg1	M1	NM-401 C2	1.15±0.33	0.7000
Arg1	M2	CNC C1	1.26±0.36	0.5300
Arg1	M2	CNC C2	0.92±0.14	0.7800
Arg1	M2	CNF C1	1.40±0.78	0.3000
Arg1	M2	CNF C2	**1.84±1.45**	**0.0160**
Arg1	M2	NM-400 C1	1.52±0.58	0.2100
Arg1	M2	NM-400 C2	1.60±0.63	0.1100
Arg1	M2	NM-401 C1	**2.49±0.95**	**0.0001**
Arg1	M2	NM-401 C2	**2.16±0.69**	**0.0007**
Cxcl10	M0	CNC C1	0.96±0.22	0.7500
Cxcl10	M0	CNC C2	1.19±0.14	0.6800
Cxcl10	M0	CNF C1	0.59±0.06	0.0720
Cxcl10	M0	CNF C2	1.11±0.35	0.7800
Cxcl10	M0	NM-400 C1	**0.41±0.08**	**0.0120**
Cxcl10	M0	NM-400 C2	0.74±0.18	0.2100

Cxcl10	M0	NM-401 C1	0.84±0.25	0.4300
Cxcl10	M0	NM-401 C2	0.95±0.07	0.7500
Cxcl10	M1	CNC C1	1.06±0.22	0.9500
Cxcl10	M1	CNC C2	1.18±0.10	0.6000
Cxcl10	M1	CNF C1	0.82±0.31	0.3200
Cxcl10	M1	CNF C2	1.74±0.20	0.0110
Cxcl10	M1	NM-400 C1	**0.53±0.13**	**0.0400**
Cxcl10	M1	NM-400 C2	0.62±0.15	0.0800
Cxcl10	M1	NM-401 C1	0.94±0.11	0.6900
Cxcl10	M1	NM-401 C2	1.12±0.27	0.9300
Cxcl10	M2	CNC C1	1.26±0.22	0.7500
Cxcl10	M2	CNC C2	1.59±0.64	0.0900
Cxcl10	M2	CNF C1	0.73±0.27	0.0910
Cxcl10	M2	CNF C2	1.21±0.42	0.6700
Cxcl10	M2	NM-400 C1	**0.50±0.13**	**0.0160**
Cxcl10	M2	NM-400 C2	0.70±0.03	0.0850
Cxcl10	M2	NM-401 C1	0.79±0.14	0.2100
Cxcl10	M2	NM-401 C2	1.16±0.36	0.9200
Cxcl9	M0	CNC C1	0.86±0.46	0.5700
Cxcl9	M0	CNC C2	1.01±0.16	0.9000
Cxcl9	M0	CNF C1	0.81±0.22	0.6300
Cxcl9	M0	CNF C2	1.12±0.55	0.8600
Cxcl9	M0	NM-400 C1	0.96±0.24	0.9000
Cxcl9	M0	NM-400 C2	0.85±0.19	0.7000
Cxcl9	M0	NM-401 C1	0.76±0.17	0.6000
Cxcl9	M0	NM-401 C2	0.97±0.41	0.8300
Cxcl9	M1	CNC C1	0.96±0.49	0.8600
Cxcl9	M1	CNC C2	1.24±0.30	0.6400
Cxcl9	M1	CNF C1	0.84±0.40	0.6300
Cxcl9	M1	CNF C2	**4.07±0.64**	**0.0000**
Cxcl9	M1	NM-400 C1	0.48±0.15	0.1400
Cxcl9	M1	NM-400 C2	0.77±0.41	0.5200
Cxcl9	M1	NM-401 C1	1.12±0.41	0.8500
Cxcl9	M1	NM-401 C2	1.22±0.55	0.6500
Cxcl9	M2	CNC C1	1.57±0.61	0.2700
Cxcl9	M2	CNC C2	1.27±0.25	0.7700
Cxcl9	M2	CNF C1	1.55±0.70	0.3300
Cxcl9	M2	CNF C2	1.33±0.30	0.7000
Cxcl9	M2	NM-400 C1	1.57±0.44	0.3400
Cxcl9	M2	NM-400 C2	1.18±0.11	0.9900
Cxcl9	M2	NM-401 C1	1.41±0.44	0.5300
Cxcl9	M2	NM-401 C2	1.62±0.53	0.2100
Ear11	M0	CNC C1	0.96±0.28	0.9600
Ear11	M0	CNC C2	1.24±0.40	0.7300
Ear11	M0	CNF C1	0.93±0.10	0.9200
Ear11	M0	CNF C2	1.40±0.42	0.4900

Ear11	M0	NM-400 C1	1.69±0.38	0.2100
Ear11	M0	NM-400 C2	1.09±0.27	0.8800
Ear11	M0	NM-401 C1	0.91±0.28	0.8800
Ear11	M0	NM-401 C2	1.81±0.41	0.1800
Ear11	M1	CNC C1	1.29±0.81	0.8800
Ear11	M1	CNC C2	1.12±0.19	0.9000
Ear11	M1	CNF C1	1.49±0.73	0.6200
Ear11	M1	CNF C2	1.78±0.79	0.3400
Ear11	M1	NM-400 C1	1.28±0.38	0.9800
Ear11	M1	NM-400 C2	1.58±0.48	0.6500
Ear11	M1	NM-401 C1	0.95±0.24	0.7200
Ear11	M1	NM-401 C2	1.45±0.74	0.6500
Ear11	M2	CNC C1	1.56±0.27	0.3900
Ear11	M2	CNC C2	1.77±0.62	0.1900
Ear11	M2	CNF C1	**2.31±0.90**	**0.0180**
Ear11	M2	CNF C2	1.79±0.20	0.2000
Ear11	M2	NM-400 C1	**3.42±0.73**	**0.0000**
Ear11	M2	NM-400 C2	**3.14±1.64**	**0.0001**
Ear11	M2	NM-401 C1	**3.98±0.95**	**0.0000**
Ear11	M2	NM-401 C2	**3.35±0.94**	**0.0000**
Il6	M0	CNC C1	0.84±0.57	0.5700
Il6	M0	CNC C2	1.16±1.58	0.7600
Il6	M0	CNF C1	0.98±0.17	0.7200
Il6	M0	CNF C2	1.64±0.43	0.8800
Il6	M0	NM-400 C1	0.53±0.35	0.3400
Il6	M0	NM-400 C2	0.99±0.95	0.6500
Il6	M0	NM-401 C1	0.85±0.55	0.8300
Il6	M0	NM-401 C2	1.73±0.83	0.6900
Il6	M1	CNC C1	0.76±0.19	0.7200
Il6	M1	CNC C2	1.17±1.11	0.9900
Il6	M1	CNF C1	1.54±1.92	0.7000
Il6	M1	CNF C2	**7.66±2.49**	**0.0000**
Il6	M1	NM-400 C1	0.35±0.08	0.3600
Il6	M1	NM-400 C2	1.11±0.78	0.9100
Il6	M1	NM-401 C1	0.61±0.33	0.7600
Il6	M1	NM-401 C2	1.81±1.98	0.3600
Il6	M2	CNC C1	0.43±0.31	0.5400
Il6	M2	CNC C2	0.57±0.40	0.7500
Il6	M2	CNF C1	1.27±0.34	0.8700
Il6	M2	CNF C2	1.36±0.63	0.7200
Il6	M2	NM-400 C1	1.61±1.44	0.5000
Il6	M2	NM-400 C2	1.17±1.14	0.8000
Il6	M2	NM-401 C1	1.83±1.06	0.2700
Il6	M2	NM-401 C2	1.30±0.49	0.6500
Mrc1	M0	CNC C1	0.71±0.20	0.4500
Mrc1	M0	CNC C2	0.77±0.21	0.5300

Mrc1	M0	CNF C1	1.46±0.87	0.4800
Mrc1	M0	CNF C2	0.60±0.23	0.3600
Mrc1	M0	NM-400 C1	**2.35±0.39**	**0.0096**
Mrc1	M0	NM-400 C2	0.57±0.21	0.3200
Mrc1	M0	NM-401 C1	2.04±0.61	0.0990
Mrc1	M0	NM-401 C2	2.13±0.55	0.0770
Mrc1	M1	CNC C1	1.46±0.75	0.3800
Mrc1	M1	CNC C2	1.08±0.22	0.8900
Mrc1	M1	CNF C1	1.20±0.36	0.8500
Mrc1	M1	CNF C2	1.70±0.91	0.1600
Mrc1	M1	NM-400 C1	1.28±0.76	0.6500
Mrc1	M1	NM-400 C2	1.09±0.14	0.9100
Mrc1	M1	NM-401 C1	1.37±0.47	0.6300
Mrc1	M1	NM-401 C2	1.36±0.69	0.6200
Mrc1	M2	CNC C1	1.30±0.69	0.7100
Mrc1	M2	CNC C2	1.65±0.53	0.2700
Mrc1	M2	CNF C1	1.90±1.05	0.1200
Mrc1	M2	CNF C2	1.08±1.05	0.9900
Mrc1	M2	NM-400 C1	1.86±0.73	0.1200
Mrc1	M2	NM-400 C2	**2.06±0.84**	**0.0460**
Mrc1	M2	NM-401 C1	**3.65±0.96**	**0.0000**
Mrc1	M2	NM-401 C2	**4.50±0.66**	**0.0000**
Nos2	M0	CNC C1	0.95±0.23	0.7800
Nos2	M0	CNC C2	1.20±0.88	0.9500
Nos2	M0	CNF C1	0.69±0.06	0.4300
Nos2	M0	CNF C2	1.48±0.69	0.3500
Nos2	M0	NM-400 C1	0.67±0.23	0.4500
Nos2	M0	NM-400 C2	0.69±0.26	0.4900
Nos2	M0	NM-401 C1	0.84±0.12	0.7500
Nos2	M0	NM-401 C2	1.73±0.64	0.2000
Nos2	M1	CNC C1	1.16±0.12	0.8400
Nos2	M1	CNC C2	1.41±0.19	0.4900
Nos2	M1	CNF C1	1.17±0.53	0.8100
Nos2	M1	CNF C2	**2.64±0.58**	**0.0008**
Nos2	M1	NM-400 C1	0.70±0.03	0.5500
Nos2	M1	NM-400 C2	0.88±0.16	0.7100
Nos2	M1	NM-401 C1	0.74±0.18	0.7000
Nos2	M1	NM-401 C2	1.16±0.38	0.6700
Nos2	M2	CNC C1	1.01±0.38	0.7200
Nos2	M2	CNC C2	1.57±1.04	0.3400
Nos2	M2	CNF C1	1.00±0.42	0.6800
Nos2	M2	CNF C2	1.95±1.07	0.0600
Nos2	M2	NM-400 C1	0.72±0.34	0.3400
Nos2	M2	NM-400 C2	0.69±0.22	0.3000
Nos2	M2	NM-401 C1	0.73±0.13	0.4500
Nos2	M2	NM-401 C2	0.70±0.34	0.4500

Supplementary Table 3. Effects on the expression of genes regulating epigenetic modifications following exposure to CNC, CNF, NM-400 and NM-401. C1: 0.15 µg/cm^2 and C2: 2.7 µg/cm^2. Significant fold changes (≥ 1.5 or ≤ 0.67, p<0.05) are indicated in bold, (n=5).

Gene symbol	Cell	Treatment	Fold changes (mean±SD)	Adj.p-value
Ash1l	M0	CNC C1	0.85±0.21	0.2600
Ash1l	M0	CNC C2	1.02±0.09	0.6400
Ash1l	M0	CNF C1	1.20±0.07	0.0130
Ash1l	M0	CNF C2	1.13±0.13	0.0870
Ash1l	M0	NM-400 C1	1.16±0.21	0.0770
Ash1l	M0	NM-400 C2	0.98±0.09	0.8300
Ash1l	M0	NM-401 C1	1.19±0.04	0.0350
Ash1l	M0	NM-401 C2	1.19±0.11	0.0280
Ash1l	M1	CNC C1	1.02±0.09	0.9000
Ash1l	M1	CNC C2	0.96±0.05	0.6800
Ash1l	M1	CNF C1	1.17±0.13	0.0870
Ash1l	M1	CNF C2	1.06±0.09	0.6100
Ash1l	M1	NM-400 C1	0.95±0.03	0.6900
Ash1l	M1	NM-400 C2	0.95±0.10	0.6600
Ash1l	M1	NM-401 C1	1.11±0.01	0.3600
Ash1l	M1	NM-401 C2	1.01±0.11	0.9300
Ash1l	M2	CNC C1	1.07±0.11	0.5300
Ash1l	M2	CNC C2	1.05±0.18	0.6700
Ash1l	M2	CNF C1	1.28±0.16	0.0026
Ash1l	M2	CNF C2	1.31±0.18	0.0008
Ash1l	M2	NM-400 C1	1.03±0.10	0.8800
Ash1l	M2	NM-400 C2	0.84±0.10	0.0950
Ash1l	M2	NM-401 C1	1.10±0.06	0.4700
Ash1l	M2	NM-401 C2	0.71±0.19	0.0022
Dnmt1	M0	CNC C1	0.71±0.19	0.2400
Dnmt1	M0	CNC C2	0.71±0.05	0.2400
Dnmt1	M0	CNF C1	0.78±0.07	0.4700
Dnmt1	M0	CNF C2	0.77±0.14	0.4400
Dnmt1	M0	NM-400 C1	1.22±0.18	0.2100
Dnmt1	M0	NM-400 C2	1.24±0.21	0.1300
Dnmt1	M0	NM-401 C1	**1.50±0.07**	**0.0035**
Dnmt1	M0	NM-401 C2	1.34±0.11	0.0230
Dnmt1	M1	CNC C1	0.83±0.10	0.6900
Dnmt1	M1	CNC C2	0.77±0.04	0.4700
Dnmt1	M1	CNF C1	0.83±0.10	0.6900
Dnmt1	M1	CNF C2	0.76±0.13	0.4500
Dnmt1	M1	NM-400 C1	0.98±0.02	0.8200
Dnmt1	M1	NM-400 C2	1.08±0.03	0.5300
Dnmt1	M1	NM-401 C1	**1.52±0.16**	**0.0025**
Dnmt1	M1	NM-401 C2	**1.56±0.21**	**0.0007**
Dnmt1	M2	CNC C1	0.94±0.39	0.6400
Dnmt1	M2	CNC C2	1.02±0.47	0.9300

Dnmt1	M2	CNF C1	1.14±0.57	0.6900
Dnmt1	M2	CNF C2	1.06±0.48	0.9500
Dnmt1	M2	NM-400 C1	0.95±0.12	0.6600
Dnmt1	M2	NM-400 C2	0.91±0.08	0.5300
Dnmt1	M2	NM-401 C1	1.48±0.03	0.0320
Dnmt1	M2	NM-401 C2	**1.85±0.25**	**0.0000**
Dnmt3a	M0	CNC C1	1.37±0.21	0.0003
Dnmt3a	M0	CNC C2	1.24±0.07	0.0300
Dnmt3a	M0	CNF C1	1.20±0.10	0.0970
Dnmt3a	M0	CNF C2	0.94±0.11	0.3600
Dnmt3a	M0	NM-400 C1	0.84±0.01	0.1000
Dnmt3a	M0	NM-400 C2	0.89±0.10	0.2100
Dnmt3a	M0	NM-401 C1	0.77±0.06	0.0180
Dnmt3a	M0	NM-401 C2	0.76±0.06	0.0081
Dnmt3a	M1	CNC C1	1.09±0.03	0.4600
Dnmt3a	M1	CNC C2	1.10±0.11	0.3500
Dnmt3a	M1	CNF C1	1.00±0.13	0.8800
Dnmt3a	M1	CNF C2	0.94±0.20	0.4500
Dnmt3a	M1	NM-400 C1	0.76±0.05	0.0200
Dnmt3a	M1	NM-400 C2	0.76±0.04	0.0130
Dnmt3a	M1	NM-401 C1	0.70±0.03	0.0031
Dnmt3a	M1	NM-401 C2	0.72±0.06	0.0024
Dnmt3a	M2	CNC C1	1.18±0.13	0.1000
Dnmt3a	M2	CNC C2	1.16±0.18	0.1700
Dnmt3a	M2	CNF C1	1.04±0.08	0.7800
Dnmt3a	M2	CNF C2	1.03±0.08	0.9100
Dnmt3a	M2	NM-400 C1	0.76±0.03	0.0160
Dnmt3a	M2	NM-400 C2	0.94±0.04	0.4400
Dnmt3a	M2	NM-401 C1	0.71±0.11	0.0028
Dnmt3a	M2	NM-401 C2	**0.56±0.03**	**0.0000**
Dnmt3b	M0	CNC C1	1.46±0.12	0.0140
Dnmt3b	M0	CNC C2	1.12±0.06	0.8900
Dnmt3b	M0	CNF C1	1.33±0.14	0.2100
Dnmt3b	M0	CNF C2	1.19±0.22	0.8800
Dnmt3b	M0	NM-400 C1	**0.64±0.18**	**0.0003**
Dnmt3b	M0	NM-400 C2	**0.66±0.08**	**0.0002**
Dnmt3b	M0	NM-401 C1	**0.63±0.04**	**0.0003**
Dnmt3b	M0	NM-401 C2	**0.62±0.09**	**0.0001**
Dnmt3b	M1	CNC C1	1.16±0.14	0.3000
Dnmt3b	M1	CNC C2	1.12±0.21	0.4700
Dnmt3b	M1	CNF C1	1.02±0.16	0.9900
Dnmt3b	M1	CNF C2	1.04±0.15	0.9100
Dnmt3b	M1	NM-400 C1	**0.60±0.10**	**0.0019**
Dnmt3b	M1	NM-400 C2	**0.58±0.05**	**0.0006**
Dnmt3b	M1	NM-401 C1	**0.62±0.08**	**0.0034**
Dnmt3b	M1	NM-401 C2	0.68±0.09	0.0059

Dnmt3b	M2	CNC C1	1.10±0.12	0.9900
Dnmt3b	M2	CNC C2	0.98±0.08	0.4000
Dnmt3b	M2	CNF C1	1.21±0.26	0.3500
Dnmt3b	M2	CNF C2	1.10±0.16	0.9100
Dnmt3b	M2	NM-400 C1	0.69±0.08	0.0031
Dnmt3b	M2	NM-400 C2	**0.56±0.06**	**0.0001**
Dnmt3b	M2	NM-401 C1	0.68±0.05	0.0024
Dnmt3b	M2	NM-401 C2	**0.56±0.08**	**0.0000**
Dot1l	M0	CNC C1	1.24±0.09	0.0790
Dot1l	M0	CNC C2	1.12±0.15	0.4700
Dot1l	M0	CNF C1	1.20±0.12	0.1800
Dot1l	M0	CNF C2	1.09±0.16	0.7000
Dot1l	M0	NM-400 C1	0.84±0.21	0.1400
Dot1l	M0	NM-400 C2	0.90±0.05	0.2900
Dot1l	M0	NM-401 C1	0.76±0.16	0.0320
Dot1l	M0	NM-401 C2	0.72±0.08	0.0100
Dot1l	M1	CNC C1	1.06±0.06	0.7300
Dot1l	M1	CNC C2	1.03±0.08	0.8800
Dot1l	M1	CNF C1	1.02±0.18	0.9200
Dot1l	M1	CNF C2	0.97±0.09	0.7800
Dot1l	M1	NM-400 C1	0.81±0.10	0.0930
Dot1l	M1	NM-400 C2	0.78±0.10	0.0360
Dot1l	M1	NM-401 C1	**0.67±0.06**	**0.0041**
Dot1l	M1	NM-401 C2	0.71±0.09	0.0059
Dot1l	M2	CNC C1	1.22±0.11	0.0930
Dot1l	M2	CNC C2	1.14±0.13	0.3800
Dot1l	M2	CNF C1	1.02±0.12	0.9800
Dot1l	M2	CNF C2	1.06±0.14	0.7500
Dot1l	M2	NM-400 C1	0.86±0.10	0.2100
Dot1l	M2	NM-400 C2	0.81±0.04	0.0970
Dot1l	M2	NM-401 C1	**0.65±0.18**	**0.0021**
Dot1l	M2	NM-401 C2	**0.51±0.05**	**0.0000**
Ezh1	M0	CNC C1	1.23±0.10	0.0110
Ezh1	M0	CNC C2	0.99±0.11	0.4400
Ezh1	M0	CNF C1	1.10±0.09	0.5900
Ezh1	M0	CNF C2	1.05±0.11	0.9800
Ezh1	M0	NM-400 C1	0.92±0.05	0.1200
Ezh1	M0	NM-400 C2	0.79±0.02	0.0004
Ezh1	M0	NM-401 C1	0.81±0.12	0.0022
Ezh1	M0	NM-401 C2	0.71±0.06	0.0000
Ezh1	M1	CNC C1	1.11±0.05	0.1700
Ezh1	M1	CNC C2	1.02±0.08	0.9100
Ezh1	M1	CNF C1	1.00±0.09	0.9400
Ezh1	M1	CNF C2	1.09±0.13	0.2300
Ezh1	M1	NM-400 C1	0.87±0.04	0.0990
Ezh1	M1	NM-400 C2	0.70±0.07	0.0000

Ezh1	M1	NM-401 C1	0.73±0.07	0.0004
Ezh1	M1	NM-401 C2	0.72±0.02	0.0001
Ezh1	M2	CNC C1	1.03±0.07	0.7600
Ezh1	M2	CNC C2	0.89±0.10	0.1400
Ezh1	M2	CNF C1	0.99±0.10	0.9200
Ezh1	M2	CNF C2	0.91±0.12	0.2100
Ezh1	M2	NM-400 C1	0.89±0.09	0.1600
Ezh1	M2	NM-400 C2	0.83±0.04	0.0180
Ezh1	M2	NM-401 C1	0.85±0.13	0.0510
Ezh1	M2	NM-401 C2	**0.52±0.05**	**0.0000**
Ezh2	M0	CNC C1	0.90±0.15	0.0930
Ezh2	M0	CNC C2	0.96±0.05	0.4900
Ezh2	M0	CNF C1	0.99±0.07	0.8400
Ezh2	M0	CNF C2	0.93±0.13	0.2900
Ezh2	M0	NM-400 C1	1.18±0.10	0.0280
Ezh2	M0	NM-400 C2	0.99±0.03	0.9000
Ezh2	M0	NM-401 C1	1.25±0.07	0.0013
Ezh2	M0	NM-401 C2	1.15±0.04	0.0350
Ezh2	M1	CNC C1	0.94±0.06	0.3900
Ezh2	M1	CNC C2	0.97±0.03	0.6700
Ezh2	M1	CNF C1	0.92±0.06	0.2400
Ezh2	M1	CNF C2	0.91±0.04	0.1500
Ezh2	M1	NM-400 C1	1.00±0.01	0.9900
Ezh2	M1	NM-400 C2	0.99±0.06	0.8400
Ezh2	M1	NM-401 C1	1.03±0.18	0.7800
Ezh2	M1	NM-401 C2	0.96±0.02	0.6800
Ezh2	M2	CNC C1	1.02±0.08	0.4500
Ezh2	M2	CNC C2	1.04±0.17	0.3400
Ezh2	M2	CNF C1	1.13±0.13	0.0170
Ezh2	M2	CNF C2	1.08±0.10	0.0990
Ezh2	M2	NM-400 C1	1.06±0.02	0.3000
Ezh2	M2	NM-400 C2	0.95±0.04	0.8700
Ezh2	M2	NM-401 C1	1.17±0.04	0.0120
Ezh2	M2	NM-401 C2	0.98±0.07	0.8800
Hdac2	M0	CNC C1	1.15±0.07	0.0110
Hdac2	M0	CNC C2	1.06±0.10	0.3600
Hdac2	M0	CNF C1	1.11±0.06	0.0460
Hdac2	M0	CNF C2	1.10±0.10	0.0850
Hdac2	M0	NM-400 C1	1.17±0.16	0.0096
Hdac2	M0	NM-400 C2	1.06±0.10	0.3500
Hdac2	M0	NM-401 C1	1.12±0.03	0.0800
Hdac2	M0	NM-401 C2	1.16±0.06	0.0096
Hdac2	M1	CNC C1	1.08±0.05	0.2100
Hdac2	M1	CNC C2	0.99±0.06	0.9100
Hdac2	M1	CNF C1	1.05±0.04	0.5700
Hdac2	M1	CNF C2	1.03±0.10	0.7600

Hdac2	M1	NM-400 C1	1.02±0.06	0.8600
Hdac2	M1	NM-400 C2	1.01±0.06	0.9100
Hdac2	M1	NM-401 C1	1.05±0.02	0.4600
Hdac2	M1	NM-401 C2	1.09±0.06	0.1500
Hdac2	M2	CNC C1	1.06±0.05	0.3800
Hdac2	M2	CNC C2	0.93±0.03	0.2600
Hdac2	M2	CNF C1	1.03±0.10	0.6900
Hdac2	M2	CNF C2	1.08±0.08	0.2100
Hdac2	M2	NM-400 C1	1.07±0.08	0.3300
Hdac2	M2	NM-400 C2	1.05±0.05	0.5000
Hdac2	M2	NM-401 C1	1.07±0.05	0.3500
Hdac2	M2	NM-401 C2	0.96±0.08	0.5700
Hdac3	M0	CNC C1	1.15±0.07	0.0035
Hdac3	M0	CNC C2	1.06±0.04	0.4900
Hdac3	M0	CNF C1	1.11±0.02	0.0490
Hdac3	M0	CNF C2	1.12±0.08	0.0190
Hdac3	M0	NM-400 C1	0.95±0.03	0.2300
Hdac3	M0	NM-400 C2	0.90±0.04	0.0150
Hdac3	M0	NM-401 C1	0.88±0.04	0.0045
Hdac3	M0	NM-401 C2	0.92±0.05	0.0350
Hdac3	M1	CNC C1	1.08±0.05	0.1000
Hdac3	M1	CNC C2	1.00±0.03	0.9400
Hdac3	M1	CNF C1	1.07±0.04	0.1600
Hdac3	M1	CNF C2	1.05±0.05	0.3600
Hdac3	M1	NM-400 C1	0.88±0.06	0.0240
Hdac3	M1	NM-400 C2	0.84±0.03	0.0014
Hdac3	M1	NM-401 C1	0.89±0.04	0.0230
Hdac3	M1	NM-401 C2	0.82±0.03	0.0002
Hdac3	M2	CNC C1	1.12±0.06	0.0120
Hdac3	M2	CNC C2	1.00±0.05	0.9100
Hdac3	M2	CNF C1	1.12±0.13	0.0150
Hdac3	M2	CNF C2	1.16±0.13	0.0014
Hdac3	M2	NM-400 C1	0.91±0.05	0.1100
Hdac3	M2	NM-400 C2	0.90±0.07	0.0370
Hdac3	M2	NM-401 C1	0.94±0.06	0.2100
Hdac3	M2	NM-401 C2	0.75±0.08	0.0000
Hdac4	M0	CNC C1	1.33±0.14	0.0003
Hdac4	M0	CNC C2	1.18±0.09	0.1400
Hdac4	M0	CNF C1	1.23±0.05	0.0210
Hdac4	M0	CNF C2	1.14±0.10	0.3200
Hdac4	M0	NM-400 C1	0.80±0.07	0.0010
Hdac4	M0	NM-400 C2	0.77±0.05	0.0002
Hdac4	M0	NM-401 C1	0.79±0.07	0.0006
Hdac4	M0	NM-401 C2	0.71±0.07	0.0000
Hdac4	M1	CNC C1	1.16±0.06	0.0330
Hdac4	M1	CNC C2	1.14±0.02	0.0800

Hdac4	M1	CNF C1	1.15±0.03	0.0370
Hdac4	M1	CNF C2	1.10±0.07	0.2100
Hdac4	M1	NM-400 C1	0.72±0.04	0.0005
Hdac4	M1	NM-400 C2	0.69±0.06	0.0001
Hdac4	M1	NM-401 C1	0.76±0.12	0.0021
Hdac4	M1	NM-401 C2	0.72±0.02	0.0001
Hdac4	M2	CNC C1	1.11±0.02	0.2100
Hdac4	M2	CNC C2	1.02±0.10	0.8500
Hdac4	M2	CNF C1	1.17±0.08	0.0290
Hdac4	M2	CNF C2	1.07±0.10	0.4600
Hdac4	M2	NM-400 C1	0.84±0.07	0.0430
Hdac4	M2	NM-400 C2	0.76±0.04	0.0010
Hdac4	M2	NM-401 C1	0.84±0.11	0.0370
Hdac4	M2	NM-401 C2	**0.56±0.07**	**0.0000**
Hdac9	M0	CNC C1	0.75±0.21	0.4000
Hdac9	M0	CNC C2	0.74±0.20	0.3600
Hdac9	M0	CNF C1	0.86±0.14	0.7500
Hdac9	M0	CNF C2	0.58±0.13	0.0870
Hdac9	M0	NM-400 C1	1.06±0.11	0.5900
Hdac9	M0	NM-400 C2	1.07±0.20	0.6400
Hdac9	M0	NM-401 C1	1.09±0.32	0.5000
Hdac9	M0	NM-401 C2	0.69±0.14	0.4000
Hdac9	M1	CNC C1	0.81±0.09	0.6400
Hdac9	M1	CNC C2	0.79±0.10	0.6000
Hdac9	M1	CNF C1	0.82±0.14	0.7700
Hdac9	M1	CNF C2	0.49±0.04	0.0510
Hdac9	M1	NM-400 C1	1.09±0.15	0.4800
Hdac9	M1	NM-400 C2	0.95±0.22	0.8200
Hdac9	M1	NM-401 C1	0.95±0.17	0.9100
Hdac9	M1	NM-401 C2	0.69±0.19	0.2300
Hdac9	M2	CNC C1	0.96±0.28	0.9100
Hdac9	M2	CNC C2	0.96±0.33	0.8900
Hdac9	M2	CNF C1	0.81±0.39	0.3300
Hdac9	M2	CNF C2	0.76±0.34	0.2300
Hdac9	M2	NM-400 C1	0.82±0.12	0.4400
Hdac9	M2	NM-400 C2	0.86±0.17	0.5700
Hdac9	M2	NM-401 C1	0.86±0.35	0.5400
Hdac9	M2	NM-401 C2	**0.54±0.10**	**0.0190**
Kat3a	M0	CNC C1	1.18±0.11	0.0280
Kat3a	M0	CNC C2	1.14±0.03	0.1300
Kat3a	M0	CNF C1	1.25±0.10	0.0018
Kat3a	M0	CNF C2	1.13±0.11	0.1900
Kat3a	M0	NM-400 C1	0.95±0.15	0.3000
Kat3a	M0	NM-400 C2	0.87±0.06	0.0190
Kat3a	M0	NM-401 C1	0.97±0.15	0.4300
Kat3a	M0	NM-401 C2	0.92±0.04	0.1000

Kat3a	M1	CNC C1	1.09±0.09	0.5700
Kat3a	M1	CNC C2	1.05±0.05	0.8800
Kat3a	M1	CNF C1	1.14±0.06	0.1400
Kat3a	M1	CNF C2	1.08±0.05	0.5500
Kat3a	M1	NM-400 C1	0.90±0.05	0.0910
Kat3a	M1	NM-400 C2	0.78±0.05	0.0006
Kat3a	M1	NM-401 C1	0.85±0.06	0.0150
Kat3a	M1	NM-401 C2	0.80±0.01	0.0008
Kat3a	M2	CNC C1	1.14±0.07	0.0510
Kat3a	M2	CNC C2	1.03±0.08	0.7800
Kat3a	M2	CNF C1	1.19±0.05	0.0086
Kat3a	M2	CNF C2	1.15±0.11	0.0430
Kat3a	M2	NM-400 C1	1.00±0.06	1.0000
Kat3a	M2	NM-400 C2	0.85±0.03	0.0290
Kat3a	M2	NM-401 C1	0.92±0.07	0.2900
Kat3a	M2	NM-401 C2	0.73±0.07	0.0001
Kat3b/Ep300	M0	CNC C1	1.28±0.14	0.0015
Kat3b/Ep300	M0	CNC C2	1.13±0.11	0.3300
Kat3b/Ep300	M0	CNF C1	1.32±0.07	0.0001
Kat3b/Ep300	M0	CNF C2	1.18±0.10	0.0800
Kat3b/Ep300	M0	NM-400 C1	0.91±0.09	0.0970
Kat3b/Ep300	M0	NM-400 C2	0.74±0.07	0.0000
Kat3b/Ep300	M0	NM-401 C1	0.88±0.04	0.0290
Kat3b/Ep300	M0	NM-401 C2	0.85±0.04	0.0070
Kat3b/Ep300	M1	CNC C1	1.15±0.06	0.1500
Kat3b/Ep300	M1	CNC C2	1.08±0.07	0.6800
Kat3b/Ep300	M1	CNF C1	1.22±0.10	0.0099
Kat3b/Ep300	M1	CNF C2	1.17±0.09	0.0800
Kat3b/Ep300	M1	NM-400 C1	0.80±0.02	0.0022
Kat3b/Ep300	M1	NM-400 C2	**0.64±0.05**	**0.0000**
Kat3b/Ep300	M1	NM-401 C1	0.88±0.01	0.0490
Kat3b/Ep300	M1	NM-401 C2	0.72±0.04	0.0000
Kat3b/Ep300	M2	CNC C1	1.19±0.03	0.0086
Kat3b/Ep300	M2	CNC C2	1.06±0.06	0.5300
Kat3b/Ep300	M2	CNF C1	1.30±0.01	0.0000
Kat3b/Ep300	M2	CNF C2	1.25±0.06	0.0008
Kat3b/Ep300	M2	NM-400 C1	0.87±0.04	0.0990
Kat3b/Ep300	M2	NM-400 C2	0.78±0.05	0.0019
Kat3b/Ep300	M2	NM-401 C1	0.94±0.07	0.4600
Kat3b/Ep300	M2	NM-401 C2	**0.59±0.07**	**0.0000**
Kat5	M0	CNC C1	1.12±0.06	0.1200
Kat5	M0	CNC C2	1.05±0.09	0.6300
Kat5	M0	CNF C1	1.23±0.05	0.0021
Kat5	M0	CNF C2	1.19±0.05	0.0120
Kat5	M0	NM-400 C1	1.26±0.06	0.0020
Kat5	M0	NM-400 C2	1.11±0.16	0.1800

Kat5	M0	NM-401 C1	1.19±0.06	0.0220
Kat5	M0	NM-401 C2	1.26±0.17	0.0024
Kat5	M1	CNC C1	1.18±0.09	0.0850
Kat5	M1	CNC C2	1.08±0.04	0.7600
Kat5	M1	CNF C1	1.21±0.07	0.0510
Kat5	M1	CNF C2	1.31±0.09	0.0012
Kat5	M1	NM-400 C1	1.21±0.04	0.0540
Kat5	M1	NM-400 C2	1.12±0.05	0.3400
Kat5	M1	NM-401 C1	1.18±0.06	0.1400
Kat5	M1	NM-401 C2	1.23±0.09	0.0190
Kat5	M2	CNC C1	1.09±0.08	0.3000
Kat5	M2	CNC C2	0.97±0.04	0.7200
Kat5	M2	CNF C1	1.20±0.17	0.0120
Kat5	M2	CNF C2	1.19±0.08	0.0150
Kat5	M2	NM-400 C1	1.33±0.17	0.0001
Kat5	M2	NM-400 C2	1.20±0.08	0.0190
Kat5	M2	NM-401 C1	1.26±0.16	0.0016
Kat5	M2	NM-401 C2	0.89±0.05	0.2100
Kat6a	M0	CNC C1	1.08±0.12	0.6100
Kat6a	M0	CNC C2	1.07±0.09	0.7200
Kat6a	M0	CNF C1	1.20±0.02	0.0290
Kat6a	M0	CNF C2	1.18±0.13	0.0430
Kat6a	M0	NM-400 C1	1.02±0.20	0.8800
Kat6a	M0	NM-400 C2	0.90±0.13	0.1000
Kat6a	M0	NM-401 C1	1.03±0.10	0.9200
Kat6a	M0	NM-401 C2	0.98±0.06	0.4500
Kat6a	M1	CNC C1	1.13±0.07	0.1200
Kat6a	M1	CNC C2	1.08±0.04	0.4000
Kat6a	M1	CNF C1	1.14±0.10	0.0790
Kat6a	M1	CNF C2	1.12±0.05	0.1300
Kat6a	M1	NM-400 C1	0.86±0.11	0.0900
Kat6a	M1	NM-400 C2	0.78±0.04	0.0052
Kat6a	M1	NM-401 C1	0.96±0.05	0.6500
Kat6a	M1	NM-401 C2	0.93±0.08	0.3500
Kat6a	M2	CNC C1	1.09±0.01	0.2100
Kat6a	M2	CNC C2	1.02±0.11	0.8100
Kat6a	M2	CNF C1	1.18±0.09	0.0190
Kat6a	M2	CNF C2	1.21±0.08	0.0049
Kat6a	M2	NM-400 C1	0.97±0.13	0.7500
Kat6a	M2	NM-400 C2	0.83±0.08	0.0160
Kat6a	M2	NM-401 C1	1.08±0.07	0.3400
Kat6a	M2	NM-401 C2	0.81±0.07	0.0130
Kat6b	M0	CNC C1	1.18±0.21	0.0300
Kat6b	M0	CNC C2	0.99±0.13	0.8300
Kat6b	M0	CNF C1	1.20±0.12	0.0170
Kat6b	M0	CNF C2	1.02±0.08	0.6300

Kat6b	M0	NM-400 C1	1.13±0.22	0.1900
Kat6b	M0	NM-400 C2	0.97±0.15	0.9600
Kat6b	M0	NM-401 C1	1.08±0.08	0.3800
Kat6b	M0	NM-401 C2	1.01±0.02	0.7000
Kat6b	M1	CNC C1	1.02±0.10	0.9700
Kat6b	M1	CNC C2	0.99±0.03	0.8800
Kat6b	M1	CNF C1	1.09±0.08	0.5200
Kat6b	M1	CNF C2	1.03±0.12	0.8900
Kat6b	M1	NM-400 C1	0.88±0.05	0.3200
Kat6b	M1	NM-400 C2	0.77±0.04	0.0220
Kat6b	M1	NM-401 C1	0.92±0.01	0.5100
Kat6b	M1	NM-401 C2	0.87±0.11	0.2100
Kat6b	M2	CNC C1	1.11±0.17	0.1800
Kat6b	M2	CNC C2	0.96±0.18	0.9500
Kat6b	M2	CNF C1	1.27±0.23	0.0021
Kat6b	M2	CNF C2	1.21±0.24	0.0160
Kat6b	M2	NM-400 C1	1.02±0.10	0.7600
Kat6b	M2	NM-400 C2	0.84±0.05	0.2900
Kat6b	M2	NM-401 C1	1.19±0.10	0.0800
Kat6b	M2	NM-401 C2	0.83±0.01	0.2100
Kdm1a	M0	CNC C1	0.71±0.25	0.2100
Kdm1a	M0	CNC C2	0.75±0.13	0.3300
Kdm1a	M0	CNF C1	0.85±0.17	0.6500
Kdm1a	M0	CNF C2	0.80±0.16	0.4700
Kdm1a	M0	NM-400 C1	1.05±0.12	0.5900
Kdm1a	M0	NM-400 C2	0.99±0.08	0.7600
Kdm1a	M0	NM-401 C1	1.04±0.01	0.4800
Kdm1a	M0	NM-401 C2	1.03±0.11	0.5000
Kdm1a	M1	CNC C1	0.82±0.09	0.7100
Kdm1a	M1	CNC C2	0.84±0.08	0.7800
Kdm1a	M1	CNF C1	0.76±0.15	0.4700
Kdm1a	M1	CNF C2	0.78±0.10	0.5300
Kdm1a	M1	NM-400 C1	0.78±0.05	0.6400
Kdm1a	M1	NM-400 C2	0.83±0.05	0.7100
Kdm1a	M1	NM-401 C1	0.91±0.08	0.8200
Kdm1a	M1	NM-401 C2	0.88±0.08	0.9100
Kdm1a	M2	CNC C1	0.94±0.39	0.6500
Kdm1a	M2	CNC C2	1.06±0.56	0.9400
Kdm1a	M2	CNF C1	1.06±0.48	0.9400
Kdm1a	M2	CNF C2	1.12±0.51	0.7000
Kdm1a	M2	NM-400 C1	0.71±0.10	0.0950
Kdm1a	M2	NM-400 C2	0.69±0.04	0.0540
Kdm1a	M2	NM-401 C1	0.89±0.05	0.6200
Kdm1a	M2	NM-401 C2	0.67±0.03	0.0870
Kdm6b	M0	CNC C1	1.38±0.11	0.0120
Kdm6b	M0	CNC C2	1.19±0.17	0.5700

Kdm6b	M0	CNF C1	1.33±0.14	0.0460
Kdm6b	M0	CNF C2	1.36±0.13	0.0230
Kdm6b	M0	NM-400 C1	0.72±0.06	0.0011
Kdm6b	M0	NM-400 C2	**0.59±0.09**	**0.0000**
Kdm6b	M0	NM-401 C1	0.70±0.09	0.0006
Kdm6b	M0	NM-401 C2	0.79±0.10	0.0032
Kdm6b	M1	CNC C1	1.15±0.14	0.5700
Kdm6b	M1	CNC C2	1.17±0.10	0.4800
Kdm6b	M1	CNF C1	1.27±0.12	0.0800
Kdm6b	M1	CNF C2	1.46±0.20	0.0004
Kdm6b	M1	NM-400 C1	**0.61±0.09**	**0.0001**
Kdm6b	M1	NM-400 C2	**0.66±0.08**	**0.0002**
Kdm6b	M1	NM-401 C1	0.80±0.12	0.0200
Kdm6b	M1	NM-401 C2	0.90±0.14	0.1300
Kdm6b	M2	CNC C1	1.16±0.09	0.2400
Kdm6b	M2	CNC C2	1.04±0.07	0.8600
Kdm6b	M2	CNF C1	1.26±0.08	0.0240
Kdm6b	M2	CNF C2	1.35±0.15	0.0015
Kdm6b	M2	NM-400 C1	0.70±0.10	0.0076
Kdm6b	M2	NM-400 C2	**0.61±0.09**	**0.0002**
Kdm6b	M2	NM-401 C1	0.85±0.12	0.1900
Kdm6b	M2	NM-401 C2	**0.58±0.05**	**0.0001**
Kmt2a	M0	CNC C1	1.43±0.14	0.0002
Kmt2a	M0	CNC C2	1.14±0.11	0.7500
Kmt2a	M0	CNF C1	1.32±0.09	0.0150
Kmt2a	M0	CNF C2	1.24±0.08	0.1400
Kmt2a	M0	NM-400 C1	0.76±0.04	0.0012
Kmt2a	M0	NM-400 C2	**0.64±0.06**	**0.0000**
Kmt2a	M0	NM-401 C1	0.72±0.02	0.0003
Kmt2a	M0	NM-401 C2	0.72±0.04	0.0001
Kmt2a	M1	CNC C1	1.13±0.02	0.2400
Kmt2a	M1	CNC C2	1.06±0.11	0.7000
Kmt2a	M1	CNF C1	1.19±0.06	0.0540
Kmt2a	M1	CNF C2	1.12±0.09	0.3400
Kmt2a	M1	NM-400 C1	**0.60±0.06**	**0.0000**
Kmt2a	M1	NM-400 C2	**0.63±0.11**	**0.0000**
Kmt2a	M1	NM-401 C1	**0.61±0.02**	**0.0001**
Kmt2a	M1	NM-401 C2	**0.60±0.05**	**0.0000**
Kmt2a	M2	CNC C1	1.23±0.12	0.0200
Kmt2a	M2	CNC C2	1.03±0.10	0.9300
Kmt2a	M2	CNF C1	1.28±0.10	0.0029
Kmt2a	M2	CNF C2	1.26±0.09	0.0065
Kmt2a	M2	NM-400 C1	**0.66±0.02**	**0.0006**
Kmt2a	M2	NM-400 C2	**0.56±0.02**	**0.0000**
Kmt2a	M2	NM-401 C1	0.68±0.03	0.0010
Kmt2a	M2	NM-401 C2	**0.50±0.03**	**0.0000**

Prmt1	M0	CNC C1	1.12±0.08	0.4600
Prmt1	M0	CNC C2	1.15±0.10	0.3300
Prmt1	M0	CNF C1	1.03±0.04	0.7800
Prmt1	M0	CNF C2	1.14±0.17	0.2300
Prmt1	M0	NM-400 C1	0.89±0.14	0.3200
Prmt1	M0	NM-400 C2	0.84±0.12	0.1800
Prmt1	M0	NM-401 C1	0.83±0.08	0.1200
Prmt1	M0	NM-401 C2	0.92±0.06	0.4300
Prmt1	M1	CNC C1	1.18±0.05	0.2300
Prmt1	M1	CNC C2	1.17±0.05	0.2300
Prmt1	M1	CNF C1	1.14±0.18	0.4000
Prmt1	M1	CNF C2	1.24±0.16	0.1200
Prmt1	M1	NM-400 C1	0.88±0.13	0.2700
Prmt1	M1	NM-400 C2	0.92±0.04	0.5000
Prmt1	M1	NM-401 C1	0.89±0.10	0.2900
Prmt1	M1	NM-401 C2	0.90±0.07	0.3300
Prmt1	M2	CNC C1	1.22±0.07	0.1400
Prmt1	M2	CNC C2	1.15±0.10	0.3500
Prmt1	M2	CNF C1	1.08±0.21	0.8500
Prmt1	M2	CNF C2	1.32±0.25	0.0370
Prmt1	M2	NM-400 C1	0.81±0.07	0.0910
Prmt1	M2	NM-400 C2	0.84±0.12	0.1000
Prmt1	M2	NM-401 C1	0.83±0.04	0.1200
Prmt1	M2	NM-401 C2	0.75±0.07	0.0320
Setd7	M0	CNC C1	1.08±0.07	0.0910
Setd7	M0	CNC C2	1.09±0.11	0.0630
Setd7	M0	CNF C1	1.08±0.06	0.1000
Setd7	M0	CNF C2	1.12±0.09	0.0099
Setd7	M0	NM-400 C1	0.87±0.05	0.0200
Setd7	M0	NM-400 C2	0.88±0.03	0.0200
Setd7	M0	NM-401 C1	0.89±0.06	0.0660
Setd7	M0	NM-401 C2	0.89±0.09	0.0460
Setd7	M1	CNC C1	1.06±0.04	0.2700
Setd7	M1	CNC C2	1.03±0.07	0.5900
Setd7	M1	CNF C1	1.04±0.04	0.5200
Setd7	M1	CNF C2	1.09±0.10	0.0800
Setd7	M1	NM-400 C1	0.84±0.02	0.0030
Setd7	M1	NM-400 C2	0.86±0.05	0.0048
Setd7	M1	NM-401 C1	0.87±0.04	0.0200
Setd7	M1	NM-401 C2	0.97±0.06	0.5700
Setd7	M2	CNC C1	1.06±0.04	0.2000
Setd7	M2	CNC C2	1.03±0.03	0.6500
Setd7	M2	CNF C1	1.05±0.07	0.3800
Setd7	M2	CNF C2	1.13±0.08	0.0065
Setd7	M2	NM-400 C1	0.86±0.05	0.0150
Setd7	M2	NM-400 C2	0.85±0.03	0.0016

Setd7	M2	NM-401 C1	0.88±0.04	0.0400
Setd7	M2	NM-401 C2	0.80±0.06	0.0001
Sirt1	M0	CNC C1	0.92±0.11	0.4000
Sirt1	M0	CNC C2	0.93±0.11	0.5100
Sirt1	M0	CNF C1	1.06±0.08	0.2400
Sirt1	M0	CNF C2	1.05±0.08	0.2600
Sirt1	M0	NM-400 C1	0.87±0.10	0.2000
Sirt1	M0	NM-400 C2	0.84±0.04	0.0850
Sirt1	M0	NM-401 C1	0.94±0.04	0.9500
Sirt1	M0	NM-401 C2	0.96±0.09	0.9800
Sirt1	M1	CNC C1	0.95±0.05	0.7900
Sirt1	M1	CNC C2	0.98±0.05	0.9300
Sirt1	M1	CNF C1	0.98±0.05	0.9100
Sirt1	M1	CNF C2	0.91±0.08	0.3400
Sirt1	M1	NM-400 C1	0.90±0.08	0.3400
Sirt1	M1	NM-400 C2	0.91±0.17	0.3200
Sirt1	M1	NM-401 C1	0.81±0.03	0.0510
Sirt1	M1	NM-401 C2	0.74±0.06	0.0025
Sirt1	M2	CNC C1	1.00±0.11	0.6700
Sirt1	M2	CNC C2	0.98±0.16	0.8400
Sirt1	M2	CNF C1	1.19±0.15	0.0006
Sirt1	M2	CNF C2	1.18±0.10	0.0012
Sirt1	M2	NM-400 C1	0.85±0.13	0.1200
Sirt1	M2	NM-400 C2	0.76±0.01	0.0022
Sirt1	M2	NM-401 C1	0.84±0.08	0.1300
Sirt1	M2	NM-401 C2	**0.60±0.05**	**0.0000**
Sirt2	M0	CNC C1	1.27±0.15	0.0001
Sirt2	M0	CNC C2	1.10±0.10	0.1700
Sirt2	M0	CNF C1	1.24±0.05	0.0003
Sirt2	M0	CNF C2	1.09±0.12	0.2100
Sirt2	M0	NM-400 C1	0.97±0.08	0.6800
Sirt2	M0	NM-400 C2	0.98±0.03	0.7300
Sirt2	M0	NM-401 C1	0.91±0.13	0.2100
Sirt2	M0	NM-401 C2	0.80±0.03	0.0029
Sirt2	M1	CNC C1	1.13±0.06	0.1600
Sirt2	M1	CNC C2	1.06±0.05	0.8200
Sirt2	M1	CNF C1	1.12±0.05	0.1900
Sirt2	M1	CNF C2	1.06±0.03	0.7000
Sirt2	M1	NM-400 C1	0.86±0.05	0.0180
Sirt2	M1	NM-400 C2	0.81±0.07	0.0013
Sirt2	M1	NM-401 C1	0.79±0.07	0.0008
Sirt2	M1	NM-401 C2	0.84±0.05	0.0038
Sirt2	M2	CNC C1	1.14±0.02	0.0300
Sirt2	M2	CNC C2	1.07±0.06	0.3800
Sirt2	M2	CNF C1	1.19±0.11	0.0032
Sirt2	M2	CNF C2	1.03±0.08	0.7600

Sirt2	M2	NM-400 C1	0.99±0.05	0.9200
Sirt2	M2	NM-400 C2	0.96±0.05	0.6000
Sirt2	M2	NM-401 C1	0.86±0.14	0.0630
Sirt2	M2	NM-401 C2	0.75±0.07	0.0002
Smyd2	M0	CNC C1	1.14±0.02	0.1900
Smyd2	M0	CNC C2	1.12±0.08	0.2700
Smyd2	M0	CNF C1	1.25±0.07	0.0099
Smyd2	M0	CNF C2	1.13±0.15	0.1500
Smyd2	M0	NM-400 C1	0.93±0.03	0.4300
Smyd2	M0	NM-400 C2	0.87±0.05	0.1600
Smyd2	M0	NM-401 C1	0.86±0.05	0.1400
Smyd2	M0	NM-401 C2	0.90±0.10	0.2400
Smyd2	M1	CNC C1	1.15±0.04	0.1800
Smyd2	M1	CNC C2	1.03±0.05	0.9100
Smyd2	M1	CNF C1	1.33±0.20	0.0020
Smyd2	M1	CNF C2	1.15±0.18	0.1700
Smyd2	M1	NM-400 C1	0.91±0.04	0.3400
Smyd2	M1	NM-400 C2	0.97±0.10	0.6600
Smyd2	M1	NM-401 C1	0.90±0.12	0.2600
Smyd2	M1	NM-401 C2	0.84±0.08	0.0970
Smyd2	M2	CNC C1	1.17±0.07	0.1100
Smyd2	M2	CNC C2	1.03±0.07	0.9100
Smyd2	M2	CNF C1	1.24±0.17	0.0400
Smyd2	M2	CNF C2	1.20±0.11	0.0910
Smyd2	M2	NM-400 C1	0.92±0.05	0.3600
Smyd2	M2	NM-400 C2	0.85±0.04	0.0930
Smyd2	M2	NM-401 C1	0.92±0.03	0.3600
Smyd2	M2	NM-401 C2	0.73±0.03	0.0059
Smyd3	M0	CNC C1	1.29±0.13	0.0025
Smyd3	M0	CNC C2	1.15±0.18	0.2400
Smyd3	M0	CNF C1	1.21±0.06	0.0430
Smyd3	M0	CNF C2	1.23±0.10	0.0270
Smyd3	M0	NM-400 C1	1.00±0.09	0.5900
Smyd3	M0	NM-400 C2	0.90±0.06	0.0800
Smyd3	M0	NM-401 C1	0.90±0.09	0.0950
Smyd3	M0	NM-401 C2	0.88±0.10	0.0360
Smyd3	M1	CNC C1	1.18±0.10	0.1200
Smyd3	M1	CNC C2	1.13±0.08	0.4200
Smyd3	M1	CNF C1	1.13±0.07	0.4300
Smyd3	M1	CNF C2	1.18±0.09	0.1400
Smyd3	M1	NM-400 C1	0.89±0.08	0.0830
Smyd3	M1	NM-400 C2	0.84±0.06	0.0130
Smyd3	M1	NM-401 C1	1.01±0.06	0.6900
Smyd3	M1	NM-401 C2	1.02±0.12	0.8300
Smyd3	M2	CNC C1	1.15±0.05	0.0540
Smyd3	M2	CNC C2	1.04±0.10	0.6600

Smyd3	M2	CNF C1	1.15±0.13	0.0690
Smyd3	M2	CNF C2	1.17±0.03	0.0330
Smyd3	M2	NM-400 C1	0.94±0.11	0.4800
Smyd3	M2	NM-400 C2	0.89±0.06	0.1500
Smyd3	M2	NM-401 C1	1.03±0.05	0.7900
Smyd3	M2	NM-401 C2	0.87±0.08	0.1200
Smyd5	M0	CNC C1	1.32±0.12	0.0048
Smyd5	M0	CNC C2	1.24±0.06	0.1000
Smyd5	M0	CNF C1	1.15±0.05	0.6700
Smyd5	M0	CNF C2	1.10±0.11	0.9400
Smyd5	M0	NM-400 C1	**0.58±0.06**	**0.0000**
Smyd5	M0	NM-400 C2	**0.61±0.09**	**0.0000**
Smyd5	M0	NM-401 C1	**0.60±0.06**	**0.0000**
Smyd5	M0	NM-401 C2	**0.61±0.03**	**0.0000**
Smyd5	M1	CNC C1	1.25±0.04	0.0400
Smyd5	M1	CNC C2	1.27±0.09	0.0160
Smyd5	M1	CNF C1	1.08±0.11	0.9100
Smyd5	M1	CNF C2	1.19±0.13	0.2300
Smyd5	M1	NM-400 C1	**0.59±0.06**	**0.0000**
Smyd5	M1	NM-400 C2	**0.59±0.07**	**0.0000**
Smyd5	M1	NM-401 C1	**0.58±0.05**	**0.0000**
Smyd5	M1	NM-401 C2	**0.53±0.05**	**0.0000**
Smyd5	M2	CNC C1	1.20±0.08	0.5900
Smyd5	M2	CNC C2	1.21±0.14	0.5800
Smyd5	M2	CNF C1	1.04±0.07	0.1600
Smyd5	M2	CNF C2	1.17±0.04	0.8800
Smyd5	M2	NM-400 C1	**0.57±0.04**	**0.0000**
Smyd5	M2	NM-400 C2	**0.55±0.08**	**0.0000**
Smyd5	M2	NM-401 C1	**0.55±0.09**	**0.0000**
Smyd5	M2	NM-401 C2	**0.42±0.03**	**0.0000**
Suv39h2	M0	CNC C1	0.91±0.08	0.0430
Suv39h2	M0	CNC C2	0.93±0.07	0.1000
Suv39h2	M0	CNF C1	1.01±0.06	0.8700
Suv39h2	M0	CNF C2	1.08±0.10	0.3400
Suv39h2	M0	NM-400 C1	1.04±0.10	0.8800
Suv39h2	M0	NM-400 C2	0.98±0.05	0.5800
Suv39h2	M0	NM-401 C1	1.07±0.04	0.4400
Suv39h2	M0	NM-401 C2	1.07±0.03	0.5300
Suv39h2	M1	CNC C1	0.99±0.02	0.9100
Suv39h2	M1	CNC C2	0.99±0.09	0.7800
Suv39h2	M1	CNF C1	0.96±0.04	0.5100
Suv39h2	M1	CNF C2	1.11±0.13	0.0790
Suv39h2	M1	NM-400 C1	1.01±0.09	0.9100
Suv39h2	M1	NM-400 C2	1.01±0.12	0.9700
Suv39h2	M1	NM-401 C1	0.97±0.07	0.7500
Suv39h2	M1	NM-401 C2	1.06±0.07	0.2900

Suv39h2	M2	CNC C1	0.95±0.05	0.5000
Suv39h2	M2	CNC C2	0.94±0.06	0.4300
Suv39h2	M2	CNF C1	1.04±0.06	0.5000
Suv39h2	M2	CNF C2	1.14±0.07	0.0170
Suv39h2	M2	NM-400 C1	1.04±0.09	0.5700
Suv39h2	M2	NM-400 C2	0.99±0.04	0.9800
Suv39h2	M2	NM-401 C1	1.02±0.04	0.6600
Suv39h2	M2	NM-401 C2	1.03±0.11	0.5500
Wdr5	M0	CNC C1	1.10±0.09	0.3000
Wdr5	M0	CNC C2	1.05±0.10	0.7600
Wdr5	M0	CNF C1	1.11±0.09	0.2300
Wdr5	M0	CNF C2	1.14±0.08	0.1000
Wdr5	M0	NM-400 C1	1.07±0.21	0.5300
Wdr5	M0	NM-400 C2	0.93±0.09	0.5000
Wdr5	M0	NM-401 C1	0.98±0.09	0.7600
Wdr5	M0	NM-401 C2	0.96±0.03	0.6300
Wdr5	M1	CNC C1	1.12±0.04	0.1900
Wdr5	M1	CNC C2	1.05±0.09	0.7000
Wdr5	M1	CNF C1	1.04±0.04	0.8100
Wdr5	M1	CNF C2	1.19±0.13	0.0300
Wdr5	M1	NM-400 C1	0.95±0.10	0.5300
Wdr5	M1	NM-400 C2	0.84±0.16	0.0320
Wdr5	M1	NM-401 C1	0.86±0.07	0.1000
Wdr5	M1	NM-401 C2	0.89±0.07	0.1900
Wdr5	M2	CNC C1	1.07±0.06	0.5200
Wdr5	M2	CNC C2	0.98±0.06	0.8100
Wdr5	M2	CNF C1	1.05±0.10	0.6100
Wdr5	M2	CNF C2	1.14±0.12	0.1200
Wdr5	M2	NM-400 C1	1.00±0.14	0.9100
Wdr5	M2	NM-400 C2	0.97±0.04	0.6400
Wdr5	M2	NM-401 C1	0.98±0.10	0.7900
Wdr5	M2	NM-401 C2	0.76±0.06	0.0038

Supplementary Table 4. Effects of nanoparticle exposure on known rRNA modifications. M1 and M2 polarized macrophages were exposed to CNF and NM-401 (high C2 dose: 2.7 µg/cm^2), respectively (n=3).

	**M1**		**M2**	
**rRNA modification**	**Control (mean±SD)**	**CNF (mean±SD)**	**Control (mean±SD)**	**NM-401 (mean±SD)**
**m^1A**	2,7 ± 0,0	2,7 ± 0,0	2,7 ± 0,1	2,7 ± 0,2
**m^6A**	2,6 ± 0,1	2,6 ± 0,0	2,6 ± 0,0	2,6 ± 0,2
**$m^{6,6}A$**	2,7 ± 0,2	2,9 ± 0,0	2,8 ± 0,1	3,0 ± 0,2
**m^5C**	4,4 ± 0,1	4,3 ± 0,2	4,4 ± 0,1	4,3 ± 0,2
**ac^4C**	2,8 ± 0,1	3,0 ± 0,0	2,8 ± 0,0	2,9 ± 0,2
**m^7G**	2,4 ± 0,0	2,5 ± 0,1	2,4 ± 0,1	2,5 ± 0,2
**m^2G**	0,9 ± 0,1	0,7 ± 0,1	0,8 ± 0,1	0,8 ± 0,1
**Y**	121,8 ± 1,9	122,0 ± 4,0	120,4 ± 1,2	119,6 ± 8,3
**m^3U**	1,3 ± 0,1	1,3 ± 0,0	1,3 ± 0,0	1,3 ± 0,1
**Am**	41,4 ± 4,2	41,1 ± 1,6	45,1 ± 5,4	44,6 ± 6,4
**Cm**	27,5 ± 2,5	27,5 ± 1,0	30,6 ± 3,0	30,2 ± 4,5
**Gm**	37,1 ± 4,5	38,3 ± 1,3	41,3 ± 4,5	40,4 ± 4,8
**Um**	23,5 ± 2,6	23,6 ± 1,0	25,0 ± 2,0	25,1 ± 4,1
